# Sequential acid/reduction response of triblock copolymeric nanomicelles to release camptothecin and toll-like receptor 7/8 agonist for orchestrated chemoimmunotherapy

**DOI:** 10.1186/s12951-022-01577-5

**Published:** 2022-08-11

**Authors:** Xiaoyan Ge, Yanyun Hao, Hui Li, Huajun Zhao, Yang Liu, Yutong Liu, Xia Li, Hongfei Chen, Jing Zou, Shiying Zhang, Lingling Huang, Gang Shan, Zhiyue Zhang

**Affiliations:** 1 NMPA Key Laboratory for Technology Research and Evaluation of Drug Products, Key Laboratory of Chemical Biology (Ministry of Education), Department of Pharmaceutics, School of Pharmaceutical Sciences, Cheeloo College of Medicine, Shandong University, 44 Wenhuaxi Road, Jinan, Shandong 250012 People’s Republic of China; 2 Institute of Immunopharmaceutical Sciences, School of Pharmaceutical Sciences, Cheeloo College of Medicine, Shandong University, Jinan, Shandong 250012 People’s Republic of China; 3 Department of Medicinal Chemistry, School of Pharmaceutical Sciences, Cheeloo College of Medicine, Shandong University, Jinan, Shandong 250012 People’s Republic of China

**Keywords:** Regulatory T cells, Chemoimmunotherapy, pH/GSH sequential response, Triblock copolymeric nanomicelles, TLR7/8 agonist

## Abstract

**Background:**

Immunosuppressive tumor immune microenvironment (TIME) lowers immunotherapy effectiveness. Additionally, low penetration efficiency and unpredictable drug release in tumor areas restrict tumor therapy.

**Methods:**

A triblock copolymeric micelle (Nano^PCPT+PIMDQ^) was developed to carry the chemotherapeutic drug camptothecin (CPT) and the TLR7/8 agonist 1-(4-(aminomethyl)benzyl)-2-butyl-1H-imidazo[4,5-c] quinoline-4-amine (IMDQ) to achieve deep tumor penetration and on-demand drug release by responding to acid and reduction stimuli sequentially. The synergistic antitumour efficacy of Nano^PCPT+PIMDQ^ was assessed both in vitro and in vivo.

**Results:**

Nano^PCPT+PIMDQ^ is composed of a hydrophilic PEG(polyethylene glycol) outer layer, an acid-sensitive EPEMA middle layer, and a drug inner core. Upon intratumoral injection, (i) Nano^PCPT+PIMDQ^ first responds to the acidic tumor microenvironment and disintegrates to PIMDQ and PCPT, penetrating deep regions of the tumor; (ii) tumor cells are killed by the released CPT; (iii) DCs are activated by PIMDQ to increase the infiltration of cytotoxic T lymphocyte (CTL); and (iv) both downregulated Foxp3^+^ Tregs by CPT and repolarized M2 macrophages by PIMDQ can relieve the TIME.

**Conclusion:**

This pH/GSH-responsive triblock polymer-drug conjugate reduces immunosuppression and enhances the infiltration of CTLs by codelivering CPT and IMDQ in a controllable manner, providing a promising platform for synergistic tumor chemoimmunotherapy.

**Supplementary Information:**

The online version contains supplementary material available at 10.1186/s12951-022-01577-5.

## Background

Immunotherapy is a promising strategy that elicits durable antitumor responses against malignant tumors by utilizing the body’s own immune system [1]. However, a durable clinical response is produced only in tumors classified as “immunogenic phenotypes” [2], which are characterized by high cytotoxic T lymphocyte (CTL) infiltration and low immunosuppressive burden (regulatory T (Treg) cells, M2 macrophages, myeloid-derived suppressor cells (MDSCs), etc.) [3–5]. Thus, converting “nonimmunogenic phenotypes” into “immunogenic phenotypes” tumors could be a viable solution to solve this issue. To improve the response rate of nonimmunogenic tumors (i.e., colorectal cancer) [6], it is urgent to simultaneously reverse the immunosuppressive state and enhance the infiltration of CTLs [7, 8]. CPT, a commonly used chemotherapeutic agent, is reported to inhibit Treg cells by reducing the expression of Forkhead box P3 (Foxp3) [9]. Emerging evidence indicates that IMDQ, as a variant of IMQ (imiquimod), promotes macrophage repolarization and stimulates dendritic cell (DC) maturation in tumors and tumor-draining lymph nodes (TDLNs), enhancing CTL infiltration and therapeutic efficacy [10, 11]. Therefore, combining CPT and IMDQ could reverse the immunosuppressive state (decreasing Treg cells and repolarizing M2 macrophages) and increase CTL infiltration, providing a platform to sensitize nonimmunogenic tumors.

Although promising, their precise delivery requires overcoming multiple barriers to penetrate deeply into tumors and achieve on-demand release, which remains challenging. Focusing on these obstacles, previous studies have reported the strategies of charge inversion [12] and size variation [13], demonstrating favorable deep tumor penetration activity. Additionally, a design with responsiveness to tumor microenvironment stimuli, such as pH and GSH, can be used to control drug release [14–16]. Notably, the GSH concentration inside cancer cells (2 − 10 × 10^−3^ M) is remarkably higher than that in normal tissue (2 − 10 × 10^−6^ M) [17, 18]. Taking advantage of these properties, a GSH-responsive strategy was adopted here to release drugs in tumor cells and optimize the therapeutic efficacy.

**Scheme 1.**
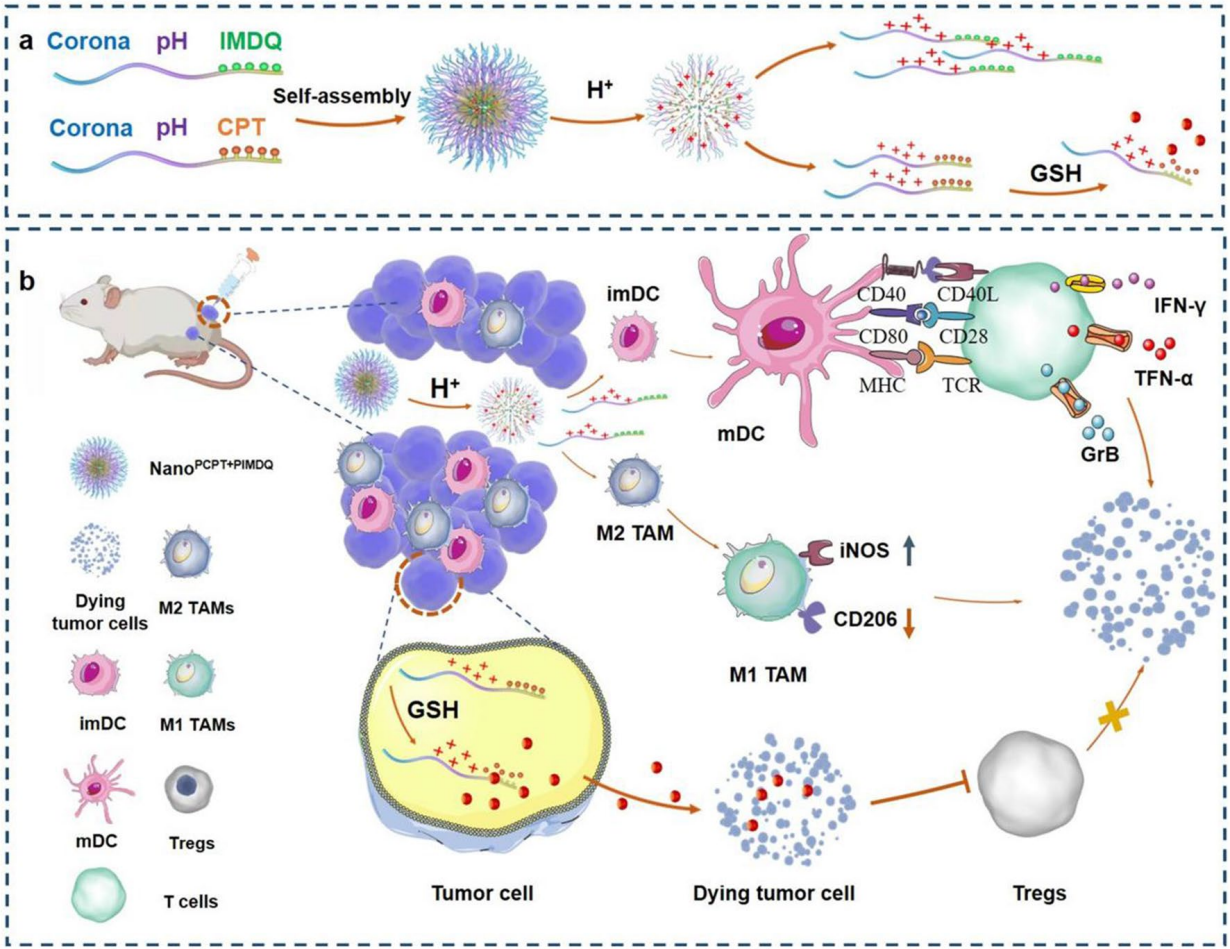
A schematic diagram of the cascade-responsive trilayer polymeric micelles (Nano^PCPT+PIMDQ^): **a** Preparation of Nano^PCPT+PIMDQ^; **b** Nano^PCPT+PIMDQ^ responds to the acidic tumor microenvironment, triggering deep tumor penetration and releasing PCPT and PIMDQ on demand. PCPT releases CPT in tumor cells in response to GSH, and the remaining CPT is released from dying tumor cells into the tumor microenvironment. Subsequently, Tregs are downregulated by CPT. **d** PIMDQ not only elicits an immune response by activating DCs but also alleviates the immunosuppressive environment by repolarizing M2 TAMs. The combination of IMDQ and CPT relieved the immunosuppressive milieu and boosted the infiltration of CTLs, thus improving colorectal cancer therapy. Figure created using BioRender.com

Herein, a cascade-responsive trilayer polymeric micelle (Nano^PCPT+PIMDQ^) was developed to codeliver CPT and TLR7/8 agonist in a controllable manner by sequentially responding to pH/GSH to trigger deep tumor penetration and on-demand release. The design of Nano^PCPT+PIMDQ^ is based on a triblock copolymer consisting of a hydrophilic poly(ethylene glycol) (PEG) segment, acid-sensitive poly(2-(N-ethyl-N-propyl amino) ethyl methacrylate) (PEPEMA) chain and hydrophobic drug segment (reduction-responsive CPT) prodrug (PCPT) chain or poly(IMDQ) (PIMDQ) chain), which are obtained by RAFT polymerization. Upon intratumoral injection (i.t.), the protonated PEPEMA becomes a hydrophilic fragment with positive charges and subsequently promotes the decomposition of Nano^PCPT+PIMDQ^ into PCPT and PIMDQ, which is beneficial for increasing cellular uptake and tumor penetration. TLR 7/8 is stimulated by PIMDQ to activate DCs, and protumoral M2 macrophages are repolarized to antitumoral M1 macrophages. Subsequently, the high GSH concentration in tumor cells triggers CPT release from PCPT, which not only provides anticancer cytotoxic effects but also decreases the expression of Foxp3 in Treg cells. Furthermore, combination with TLR7/8 agonists elicits a robust antitumor CTL response in primary and distal tumors (Scheme 1).

## Results and discussion

### Synthesis and characterization of polymers

**Fig. 1:**
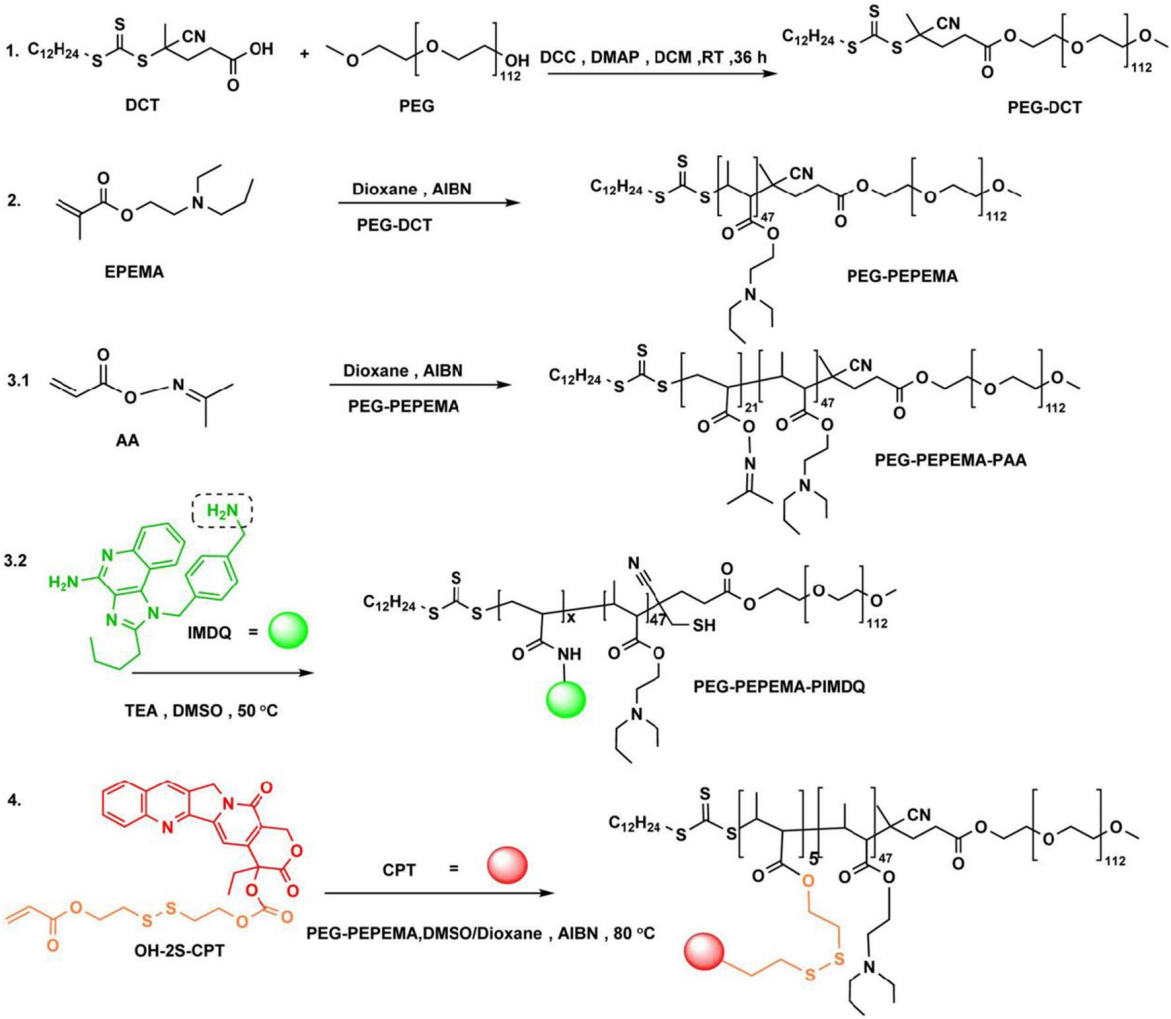
The synthetic route of PEG-PEPEMA-PCPT and PEG-PEPEMA-PIMDQ

Reversible addition-fragmentation chain transfer (RAFT) polymerization was used to prepare polymers, and the detailed synthetic route is illustrated in Fig. 1. Prior to polymerization, monomers of AA (acryloyl acetone oxime), EPEMA (2-(*N*-ethyl-*N*-propyl amino) ethyl methacrylate), and OH-2S-CPT (reduction-responsive CPT monomer) were successfully synthesized (Additional file 1: Figs. S2–S5). PEG-DCT (polyethylene glycol macro-chain transfer agent) was synthesized and utilized to polymerize EPEMA, yielding the diblock polymer PEG-PEPEMA (Additional file 1: Figs. S6, S7). The degree of polymerization (DP) was calculated to be 47 (Additional file 1: Fig. S7). Subsequently, the triblock polymeric prodrug (PEG-PEPEMA-PCPT) was composited using PEG-PEPEMA as a macro-chain transfer agent (macro-CTA) and 2,2′-azobis(2-methylpropionitrile) (AIBN) as an initiator. The resulting DP was calculated to be 5 (Additional file 1: Fig. S8). Moreover, to obtain the IMDQ-conjugated triblock polymer PEG-PEPEMA-PIMDQ, a two-step reaction was needed. First, the triblock copolymer PEG-PEPEMA-PAA was prepared using PEG-PEPEMA as a micro-CTA and AIBN as an initiator, and the DP was 21 (Additional file 1: Fig. S9). The monomer of AA in PEG-PEPEMA-PAA provided a platform for ligating amino group-containing compounds (i.e., IMDQ). Herein, IMDQ was conjugated to the main chain of the polymer by substituting AA, yielding an IMDQ-conjugated triblock polymer PEG-PEPEMA-PIMDQ (Additional file 1: Fig. S10). The grafting rates of CPT and IMDQ were calculated to be 34.09 ± 0.46% and 13.62 ± 0.19%, respectively, by UV–vis measurements (Additional file 1: Figs. S11, S12).

### Preparation and characterization of Nano^PCPT+PIMDQ^

**Fig. 2:**
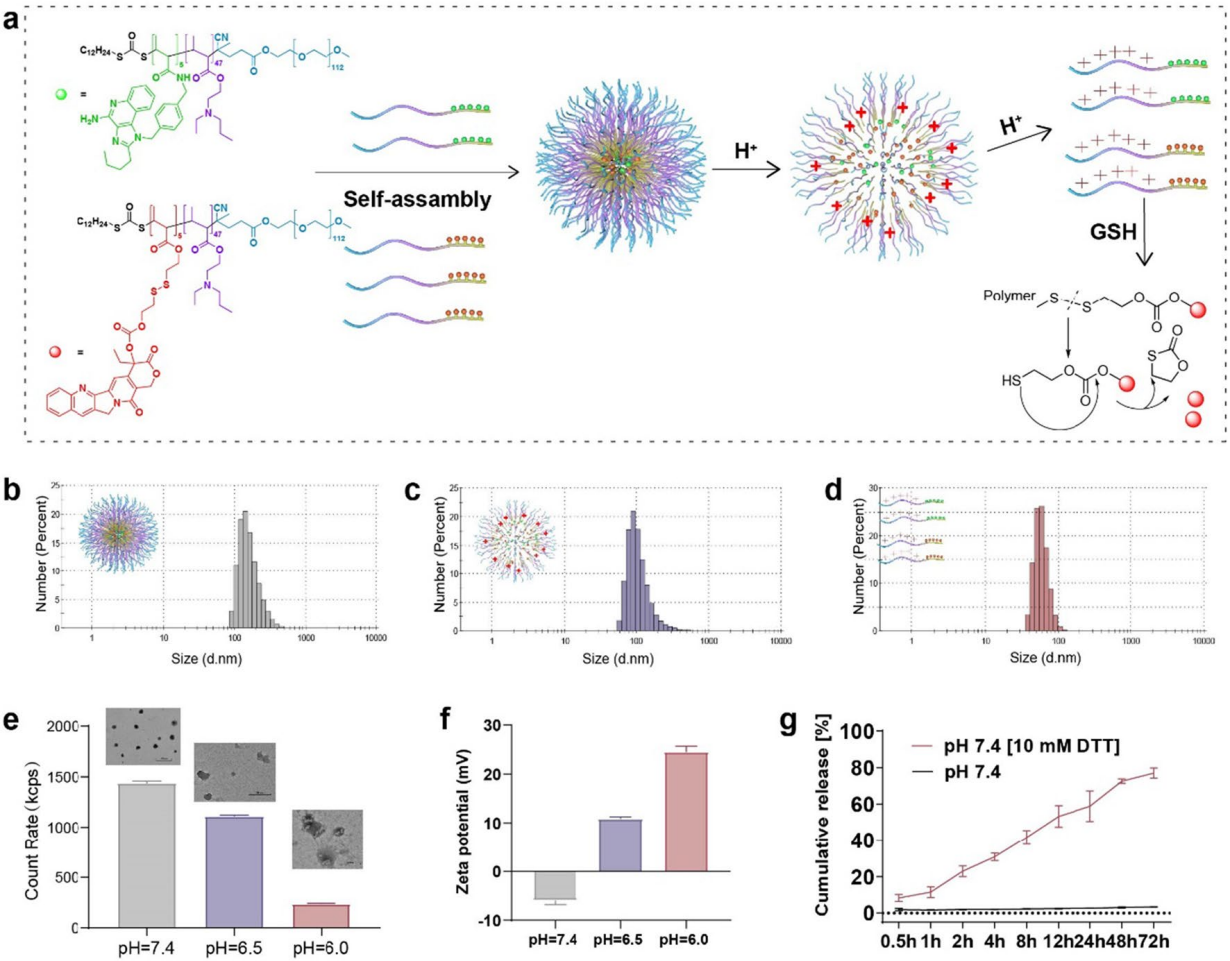
Physicochemical characterization of Nano^PCPT+PIMDQ^. **a** Schematic diagram of the structure, self-assembly and disassembly of Nano^PCPT+PIMDQ^. **b**, **d** DLS results showing the hydrodynamic diameter of Nano^PCPT+PIMDQ^ at pH **b** 7.4, **c** 6.5, and **d** 6.0. **e** Count rate of Nano^PCPT + PIMDQ^ accompanied by TEM images at pH 7.4 (bar: 500 nm), pH 6.5 (bar: 100 nm), and pH 6.0 (bar: 100 nm). **f** Zeta potentials of Nano^PCPT+PIMDQ^ under various pH conditions. **g** DTT-triggered CPT release from Nano^PCPT^ in vitro. Data are shown as the mean ± standard deviation (SD) (n = 3). *p < 0.05, **p < 0.01, ***p < 0.001

The preparation process, pH responsiveness and drug release mechanism of Nano^PCPT+PIMDQ^ are illustrated in Fig. 2a. The structure of EPEMA changed to a hydrophilic quaternary amine in the acidic tumor milieu, allowing for charge reversal and dispersion of Nano^PCPT+PIMDQ^. These changes not only enhanced the cellular uptake of the conjugate but also increased drug penetration into tumors. To verify our hypothesis, we studied the effect of pH on the size and morphology of Nano^PCPT+PIMDQ^. As illustrated in Fig. 2b–d**,** the hydrodynamic particle size of Nano^PCPT+PIMDQ^ at pH 7.4, 6.5, and 6.0 determined by dynamic light scattering (DLS) was 172.43 ± 9.57 nm, 112.83 ± 6.41 nm, and 52.78 ± 5.41 nm, respectively, with a polydispersity index (PDI) of 0.22 ± 0.00, 0.21 ± 0.01, and 0.76 ± 0.01. Transmission electron microscopy (TEM) images obtained under different pH conditions indicated the pH-induced dissociation of Nano^PCPT+PIMDQ^. Specifically, the morphology of Nano^PCPT+PIMDQ^ was spherical at pH 7.4 with a size of 100–130 nm, tended to be irregular at pH 6.5, and completely collapsed at pH 6.0, further revealing the pH dependence of its morphology. The count rate, representing the average scattering intensity of samples [19], decreased as the pH decreased, especially at pH 6.0, indicating the disassembly of Nano^PCPT+PIMDQ^ (Fig. 2e). To confirm the charge reversal induced by pH, the zeta potential of Nano^PCPT+PIMDQ^ was investigated at different pH values. The zeta potentials of Nano^PCPT+PIMDQ^ reversed from − 5.95 ± 0.87 mV at pH 7.4 to 10.87 ± 0.40 mV at pH 6.5 and 24.53 ± 1.17 mV at pH 6.0 (Fig. 2f).

The release kinetics of free CPT in vitro verified the reduction-sensitive release characteristics of nanomicelles designed to control drug release and avoid the toxicity of chemotherapy to normal cells [20]. The samples were dialyzed against various buffered medium in the absence or presence of dithiothreitol (DTT, 10 mM) to study whether the reduction circumstances influenced the CPT release characteristics of Nano^PCPT^. Negligible CPT was released from Nano^PCPT^ without DTT, no matter under which pH conditions the samples are. However, free drug was constantly released from Nano^PCPT^ if they were immersed into 10 mM DTT solution under different pH (7.4, 6.5, and 5.5), indicating that the release of drug is dependent on the reduction condition, not or a little bit dependent on the pH conditions in the tumor microenvironment (Fig. 2g and Additional file 1: Fig. S13). Altogether, the combined results revealed that Nano^PCPT+PIMDQ^ exhibited sequential pH/GSH responsiveness, laying the groundwork for our subsequent research.

### In vitro cellular uptake and cytotoxicity of nanomicelles

**Fig. 3:**
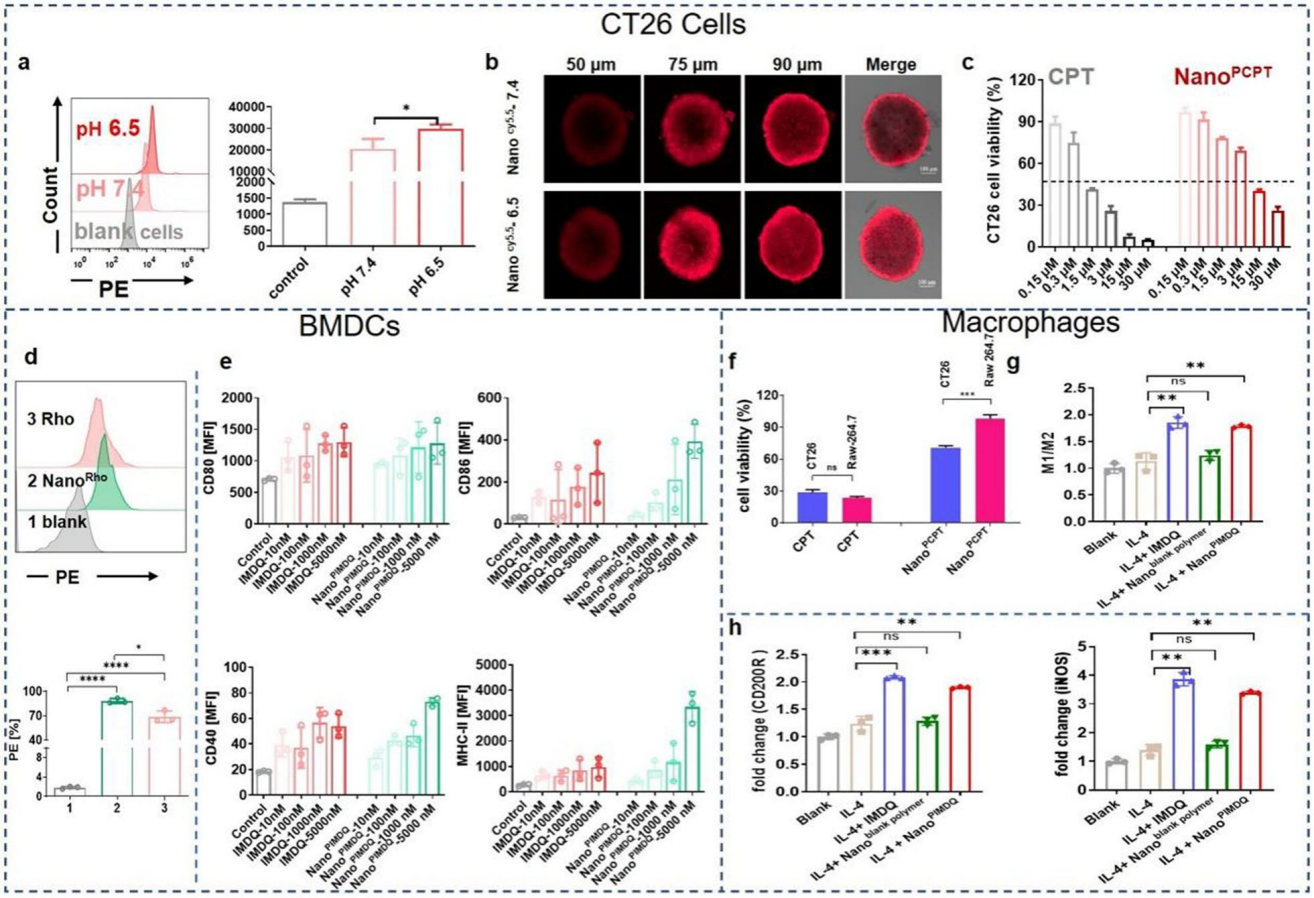
The acidic environment improved the cellular uptake efficiency of nanomicelles; nanomicelles selectively killed CT26 cells and effectively induced activation of immune cells in vitro. **a** In vitro CT26 cell uptake of nanomicelles under different pH conditions. **b** CLSM images of CT26 MCSs incubated with Nano^cy5.5^ under different pH conditions (Cy5.5-equivalent dose of 2 μg·mL^−1^). **c** CCK-8 was used to detect the vitality of CT26 cells incubated with Nano^PCPT^ (n = 6). **d** In vitro BMDC uptake using Rho as a trace at 24 h (n = 3). **e** In vitro BMDC maturation induced by Nano^PIMDQ^ and the FlowJo quantification of CD40, CD80, CD86, and MHC-II gated on CD11c^+^ cells (n = 3). **f** A CCK-8 assay was used to detect/compare the viability of CT26 cells and macrophages incubated with Nano^PCPT^ (n = 5). **g**, **h** Flow cytometric analysis results of macrophage repolarization remodeling experiment by RAW264.7 cells in vitro (n = 3). Data are represented as the mean ± SD. *p < 0.05, **p < 0.01, ***p < 0.001, ****p < 0.0001

Although CPT has an excellent curative effect, its clinical application is limited due to its low water solubility and high toxicity [21]. Traditional nano-formulations can effectively improve the in vivo pharmacokinetics of CPT, but the physical encapsulation of CPT will still cause damage to the body due to quick leakage in systemic circulation [22]. Covalent ligation to polymeric carriers might offer chemically better-defined alternatives to physical encapsulation technology. Studies have shown that the positive charge of nanomedicine is beneficial to cellular uptake [23]. To evaluate the effects of various pH levels on the cellular uptake of nanomicelles, 5(6)-TAMRA cadaverine (Rho) was used to label nanomicelles, yielding Nano^Rho^. When incubated with CT26 cells at pH = 6.5, Nano^Rho^ showed a higher mean fluorescence intensity (MFI) than that at pH = 7.4 (*p < 0.05). This result was attributed to the charge reversal from negative to positive of Nano^Rho^ at pH 6.5 (Fig. 3a), which was beneficial to enhance the uptake by tumor cells. To evaluate the tumor penetration ability of Nano^PCPT+PIMDQ^ by 3D multicellular spheroids (MCSs) of CT26 cells, cy5.5 was chosen instead of CPT or IMDQ to label triblock polymeric micelles, yielding Nano^cy5.5^. Confocal laser scanning microscopy (CLSM) showed that more Nano^cy5.5^ penetrated the interior zone of MCSs at pH 6.5 after 6 h of incubation than at pH 7.4 (Fig. 3b), which was related to the charge reversal and gradual dispersion of Nano^PCPT+PIMDQ^ in the acidic tumor microenvironment.

The achievement of charge reversal from negative to positive in the nanomicelles was merely one of the requirements for their efficacy. Subsequently, the cytotoxicity of Nano^blank polymer^ against CT26 cells was assessed by a CCK-8 assay. According to studies, zwitterionic polymers are neutral and thus have low cytotoxicity [24]. Negligible cytotoxicity was observed when the concentration of Nano^blank polymer^ reached 500 μg·mL^−1^ (Additional file 1: Fig. S14), indicating that it was cytocompatible at a given concentration range. It is worth noting that the concentration of GSH in tumors (2–10 × 10^–3^ M) is reported to be 1000 times higher than that in normal cells (2–10 × 10^–6^ M) [25]. The in vitro release of CPT demonstrated the reduction-triggered ability of Nano^PCPT^. To further confirm the reduction-triggered specific drug release in tumor cells, we investigated the toxicity of free CPT and Nano^PCPT^ to tumor cells (CT26 cells) and normal cells (RAW 264.7 cells) (equal to 3 μM CPT), respectively. As expected, free CPT had equal toxicity to RAW 264.7 cells and CT26 cells, while the viability of macrophages incubated with Nano^PCPT^ was lower than that of CT26 cells (***p < 0.001) (Fig. 3f). Additionally, we explored the toxicity of various concentrations of Nano^PCPT^ against CT26 cells. The cell viability exhibited a dose-dependent effect, and the half-maximal inhibitory concentration of the Nano^PCPT^ group determined to be the CPT-equivalent concentration was 8 μM (Fig. 3c). The results indicated that the carrier, triblock polymer, had high biocompatibility, and Nano^PCPT^ had excellent reduction-induced drug release performance against tumor cells rather than normal cells. Therefore, compared with other CPT nanocarriers, our carrier could be taken up by cells at tumor sites and had a powerful specific killing effect on tumor cells.

### In vitro immune stimulation evaluation

The ability of Nano^PIMDQ^ to activate bone marrow-derived dendritic cells (BMDCs), which are the most effective antigen-presenting cells, was determined by activation assays. For this purpose, either IMDQ or Nano^PIMDQ^ was pulsed with BMDCs, and the extent of DC activation was quantified by flow cytometric measurement of the surface expression of MHC-II and the costimulatory molecules CD80, CD86, and CD40. The intracellular uptake results (Fig. 3d and Additional file 1: Fig. S15) indicated that Rho had a stronger fluorescent signal than Nano^Rho^ after a short period of incubation. The fluorescence of Nano^Rho^ was stronger after 24 h of incubation, whereas that of free Rho was comparatively weaker. That might be due to the fact that free Rho, as a small molecule, was quickly taken up but also quickly pumped out by cells. However, Nano^Rho^, with a certain size, was gradually more taken up by BMDCs over time, which was conducive to exerting efficacy. Furthermore, BMDCs activation experiments (Fig. 3e) showed that the upregulation of MHC-II, CD80, CD40 and CD86 was dose-dependent on both the IMDQ and Nano^PIMDQ^. BMDCs expressed more MHC-II, CD40, and CD86 after treatment with Nano^PIMDQ^ than that treatment of equal IMDQ (5000 nM), mainly due to the difference in cellular uptake levels of IMDQ. Therefore, the results indicated that Nano^PIMDQ^ was still capable of activating DCs, even to a greater extent than the equivalent amount of IMDQ under the experimental conditions.

Tumor-associated macrophages (TAMs) are the major innate immune cells, accounting for approximately 50% of human tumor cells, and thus play vital roles in antitumor immunotherapy [26]. TAMs assume two opposing phenotypes: antitumoral M1 macrophages and protumoral M2 macrophages. It was reported that TAMs predominantly exhibit M2-type functions promoting intratumoral infiltration of Treg cells, thus contributing to T-cell dysfunction [27]. Therefore, reprogramming M2 macrophages into M1 macrophages could be a promising therapeutic strategy [28, 29]. Here, the effect of free IMDQ and Nano^PIMDQ^ on macrophage repolarization was investigated with IL4-pretreated Raw264.7 cells as a model of protumoral M2 macrophages. CD200R was used to mark M2 macrophages, and iNOS was used to mark M1 macrophages. As shown in Fig. 3g, h, flow cytometric analysis first indicated that Nano^blank polymer^ (without IMDQ conjugation) did not affect the inflammatory responses, suggesting that these nanomicelles were inherently nonimmunogenic under the experimental conditions. Second, both free IMDQ and Nano^PIMDQ^ treatment induced a dramatic increase in the percentage of M1 macrophages. Briefly, Nano^PIMDQ^ notably reprogrammed M2 macrophages to M1 macrophages to alleviate the immunosuppressive tumor microenvironment. Simultaneously, the percentage of M2 macrophages also slightly increased, which could result from a compensatory immune response. Thus, in our work, IMDQ-ligated nanomicelles are sufficiently capable of activating innate immune responses as well as remodeling macrophages to antitumor M1 phenotypes.

### Spatiotemporal biodistribution of Nano^cy5.5^ in vivo

**Fig. 4:**
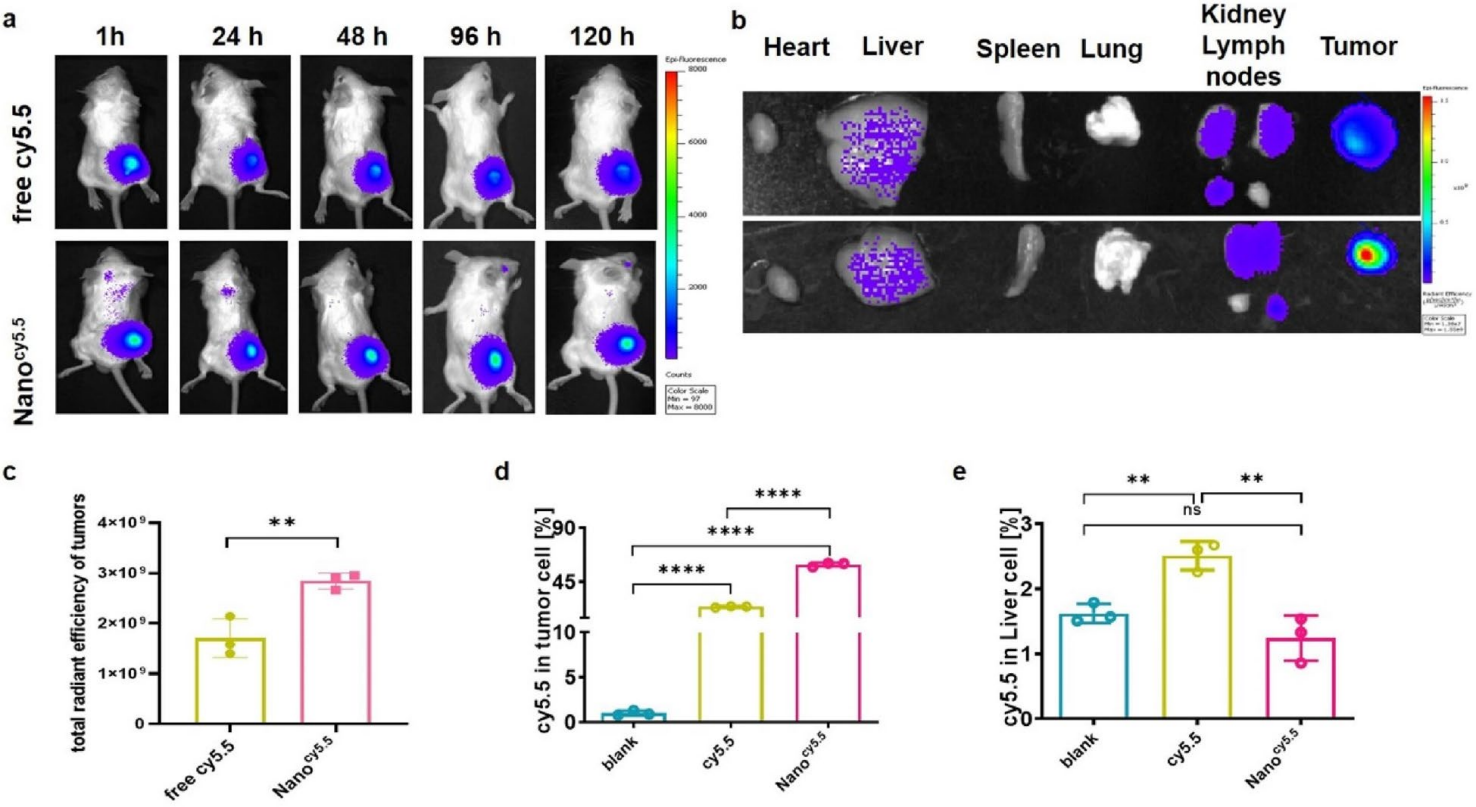
Nanomicelles increased the retention of drugs in tumor tissue and tumor cells. **a** Live imaging of BALB/c mice. **b** Ex vivo imaging of isolated organs in BALB/c mice. **c** Fluorescence quantification value in isolated tumors (n = 3). **d** Fluorescence quantification value in tumor cells and **e** in hepatocytes. Data are shown as the mean ± SD. *p < 0.05, **p < 0.01, ***p < 0.001, ****p < 0.0001

The tissue distribution and tumor retention of triblock copolymeric nanomicelles were evaluated in CT26 tumor-bearing BALB/c mice. Nanomicelles were labeled with cy5.5-amine, injected intratumourally and subsequently imaged using an in vivo imaging system (IVIS) at different time intervals post-injection. I.t. injection of Nano^cy5.5^ yielded a strong and long-term persisting fluorescent signal in the tumor sites compared with that of free Cy5.5. This finding was further confirmed by the increased cy5.5 signal in isolated tumor tissues treated with Nano^cy5.5^ (Fig. 4a–c). To observe the cellular uptake in detail, cancer cells and hepatocytes were isolated separately. Notably, the uptake of Nano^cy5.5^ in tumor cells was higher (****p < 0.0001) than that of free cy5.5, while it was lower (**p < 0.01) in hepatocytes, according to flow cytometry analysis (Fig. 4d, e). We speculate that the nanomicelles will disintegrate at the tumor site to achieve better tumor penetration, and the disintegration of the drug with a positive charge increased the adsorption of the cells, which induced our nanomicelles to have a longer retention effect after i.t. injection than the free drug. In summary, our nanomicelles have a longer retention effect in tumors, which was conducive to minimizing systemic toxicity.

### In vivo antitumor efficacy

**Fig. 5:**
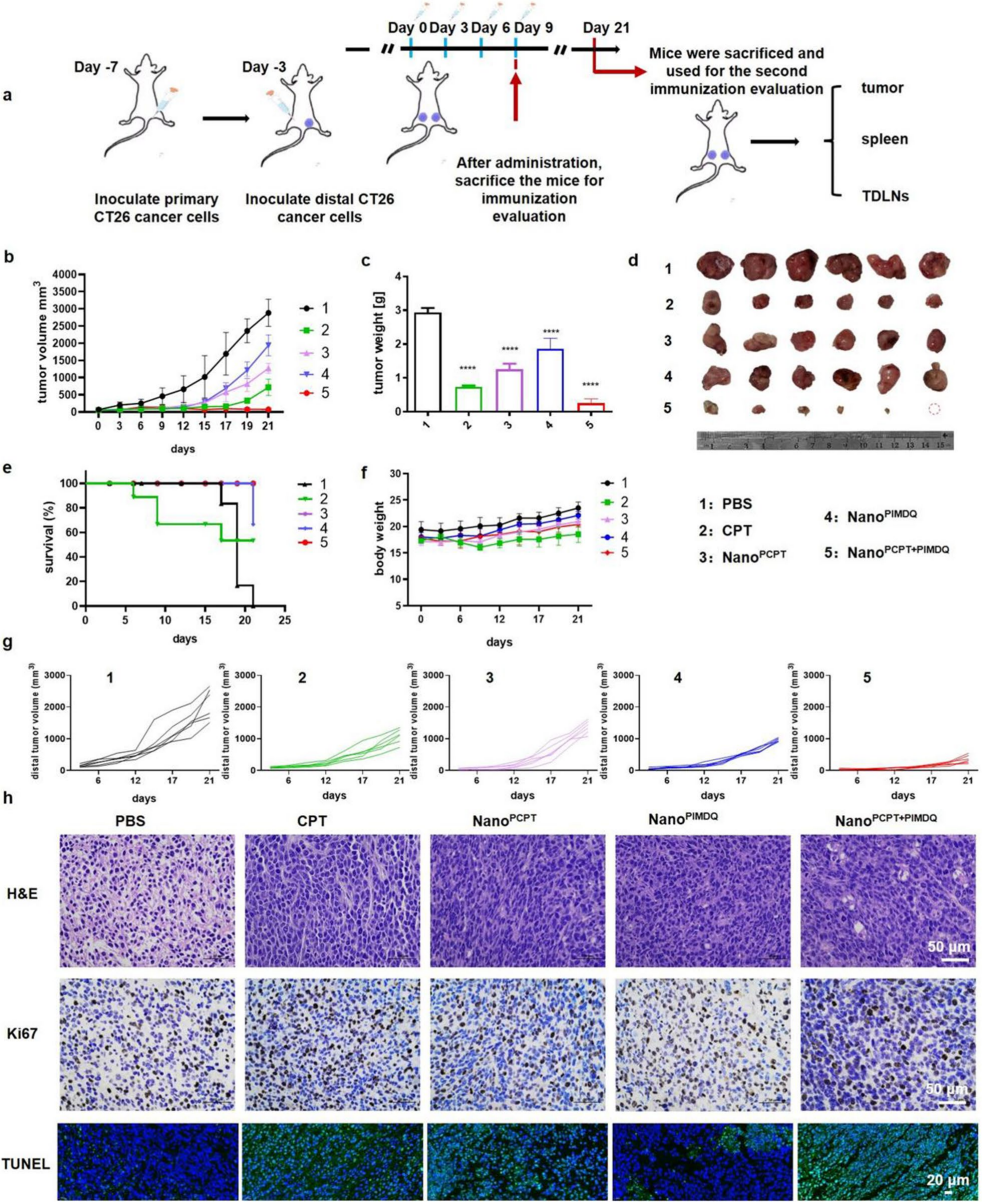
Nano^PCPT+PIMDQ^ improved antitumor efficiency in vivo. **a** Antitumor experimental design timeline of CT26-bearing BALB/c mice. **b** Proximal tumor growth curves of mice following treatment with PBS, CPT, Nano^PCPT^ and Nano^PCPT+PIMDQ^ (n = 6). **c** Weight and **d** photograph of proximal tumors harvested from the mice on Day 21 (n = 6). **e** Kaplan–Meier curves of the mice following different treatments. **f** Body weight change profile of mice during the treatment period (n = 6). **g** Distal tumor growth profiles of the mice following different treatments (n = 6). **h** H&E, Ki67, and TUNEL staining images of tumor slides collected from CT26-bearing BALB/c mice after treatment (H&E 400 × , Ki67 50 × , and TUNEL 40 ×). Data are shown as the mean ± SD. *p < 0.05, **p < 0.01, ***p < 0.001

Encouraged by the positive antitumor activity and enhanced immune responses of nanomicelles in vitro, we assessed the therapeutic potential of nanomicelles in vivo. The antitumor efficacy of nanomicelles was evaluated using bilateral CT26 cell-bearing BALB/c mice, where the tumor growing on the right side of the back of BALB/c mice was the primary tumor and that on the left was the distal tumor. When the primary tumor volume reached 50–60 mm^3^, mice were randomly grouped (n = 6) and then treated with PBS, CPT, Nano^PCPT^, Nano^PIMDQ^, or Nano^PCPT+PIMDQ^ intratumorally at equivalent doses of CPT (10 mg·kg^−1^) and IMDQ (0.5 mg·kg^−1^) according to the design schedule shown in Fig. 5a. The tumor volume was monitored using calipers, and the tumors from each treatment group were collected and weighed at the end of the survival study. Nano^PCPT+PIMDQ^ generated the most substantial inhibition in both primary and distant tumors, as shown in Fig. 5b–g, which demonstrated that the combination of CPT-mediated chemotoxicity and Treg cells inhibition, together with TLR agonist-induced immune responses, might inhibit tumor growth synergistically. Images of primary excised tumors indicated that one out of six tumors were completely eradicated after treatment with the combined CPT and IMDQ therapies. In addition, no significant changes in body weight were observed, and the Kaplan–Meier curve displayed improved survival of the mice treated with Nano^PCPT+PIMDQ^.

To further evaluate the antitumor efficacy of Nano^PCPT+PIMDQ^ in vivo, Ki67, H&E, and TUNEL stains were applied (Fig. 5h). The Ki67 staining images indicated less proliferation in the Nano^PCPT+PIMDQ^ group. The H&E staining images demonstrated that the Nano^PCPT+PIMDQ^ group had extensive cell necrosis. The TUNEL staining images showed the presence of multiple apoptotic cells in the Nano^PCPT+PIMDQ^ group. In summary, compared with the control groups, Nano^PCPT+PIMDQ^ exhibited outstanding therapeutic efficacy and prolonged survival in CT26-bearing mice. There was no obvious tissue damage in the Nano^PCPT+PIMDQ^-treated group (Additional file 1: Fig. S17).

### In vivo exploration of the antitumor immune mechanism

**Fig. 6:**
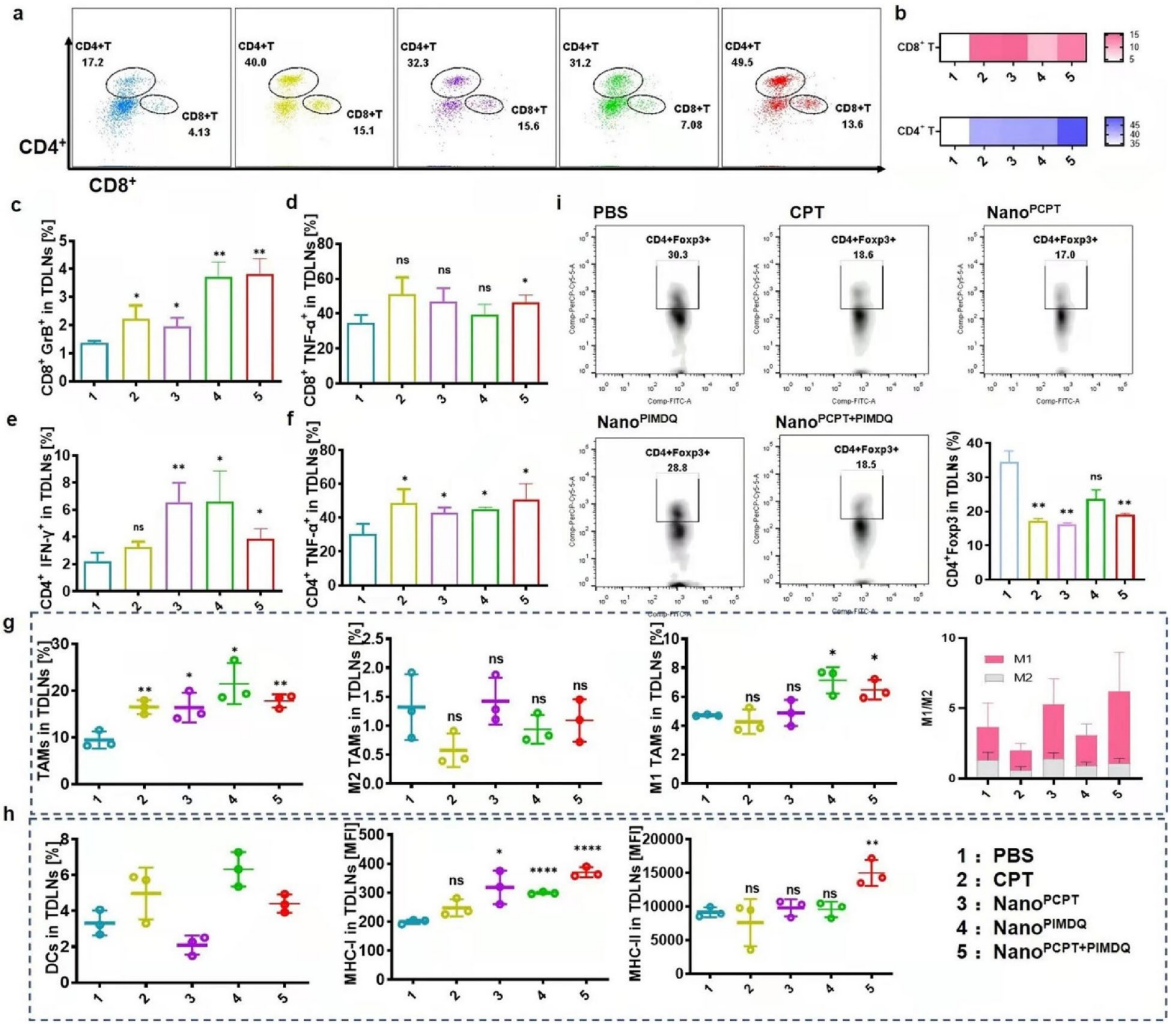
Nano^PCPT+PIMDQ^ enhanced antitumor immunity in TDLNs. **a**–**i** Flow cytometry quantification of lymphocytes in TDLNs from mice treated with various groups of samples. **a**, **b** Tabulated flow cytometric data for CD8^+^ T and CD4^+^ T cells expressed in TDLNs on the ninth day (n = 3). **c**, **d** Flow cytometry analysis of GrB^+^ and TNF-α^+^ CD8^+^ T cells in TDLNs (n = 3). **e**, **f** Flow cytometry analysis of IFN-γ^+^ and TNF-α^+^ CD4^+^ T cells in TDLNs (n = 3). **g** Flow cytometry analysis of total macrophages, M2 macrophages (CD206), M1 macrophages (iNOS) and the M1/M2 ratio in TDLNs. **h** The percentage of CD11c^+^ DCs and MFI of MHC-I and MHC-II on CD11c^+^ DCs in TDLNs from treated mice were analyzed by flow cytometry. **i** Foxp3^+^ cells gated on CD4^+^ cells collected in TDLNs. *p < 0.05, **p < 0.01, ***p < 0.001

TLR agonists can promote TLR recognition of pattern-associated molecular patterns to initiate innate immunity [30, 31]. Innate immune cells are then recruited to present the antigen, eventually priming and boosting adaptive immunity [32, 33]. These complicated steps have emphasized the critical roles of macrophages, DCs and T cells [34]. Consequently, the regulation of immune cells by Nano^PCPT+PIMDQ^ was further studied in vivo. After four administrations, the mice were sacrificed in each group, and the lymphocytes of spleens and TDLNs were harvested to evaluate the immune response. It has been reported that most of the T cells in tumors are derived from TDLNs, followed by their expansion in tumor tissue [35]. Thus, the elevated percentage of CD8^+^ T cells in TDLNs revealed that Nano^PCPT+PIMDQ^ might increase T-cell activity at the tumor site. In addition, CD8^+^ T cells showed upregulated protein expression of granulomycin B (GrB) and tumor necrosis factor α (TNF-α) upon treatment with Nano^PCPT+PIMDQ^. Nano^PCPT+PIMDQ^ also elicited a higher percentage of interferon-γ (IFN-γ)-producing CD4^+^ T cells. These results indicated that Nano^PCPT+PIMDQ^ enhanced antigen presentation and promoted T-cell effector function (Fig. 6a–f). In mice receiving Nano^PCPT+PIMDQ^, the total percentage of macrophages (F4/80^+^ cells) was upregulated (Fig. 6g). In particular, although the amount of M2 macrophages (CD206^+^) did not change obviously, the proportion of M1 macrophages (iNOS^+^) and the M1/M2 ratio were increased, indicating that macrophages had transformed from M2 to M1-like macrophages. In addition, MHC-I and MHC-II were expressed to a greater extent on the surface of DCs treated with Nano^PCPT+PIMDQ^ than on the surface of DCs treated with control agents (Fig. 6h). Moreover, the number of Treg cells (CD4^+^Foxp3^+^ T cells) decreased in the mice receiving the CPT-containing treatment, demonstrating the Treg inhabitation (Fig. 6i).

**Fig. 7:**
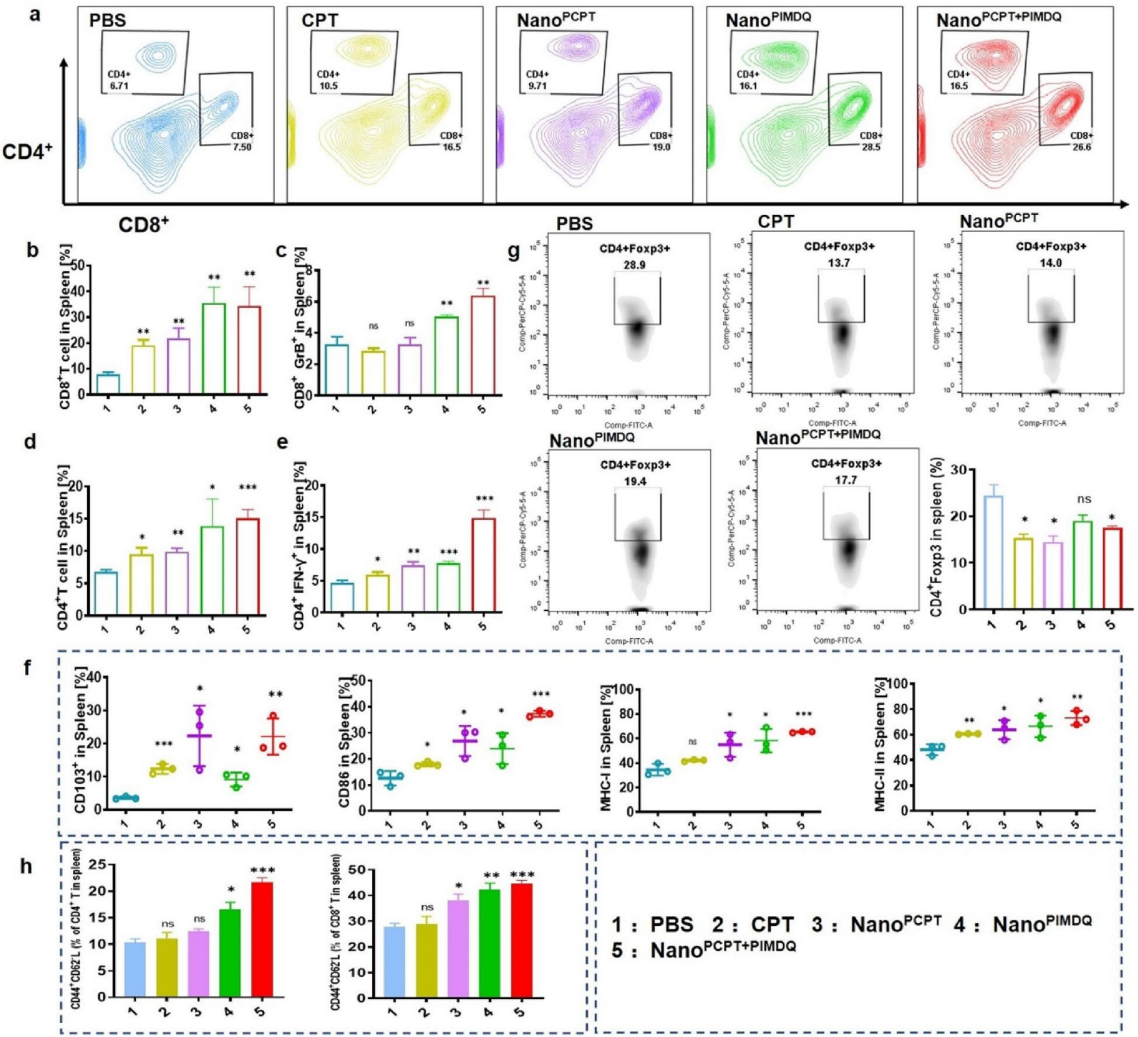
Nano^PCPT+PIMDQ^ enhanced antitumor immunity in spleens. **a**–**h** Flow cytometry quantification of lymphocytes in spleens from mice treated with various groups of samples. **a** Percentage of CD8^+^ and CD4^+^ T cells harvested in spleens on the ninth day (n = 3). **b**, **c** The frequency of CD8^+^ T cells and the expression of GrB in CD8^+^ T cells from spleens (n = 3). **d**, **e** The frequency of CD4^+^ T cells and the expression of IFN-γ in CD4^+^ T cells from spleens (n = 3). **f** Flow cytometry analysis of CD103^+^CD11c^+^, CD86^+^CD11c^+^, MHC-I^+^CD11c^+^, and MHC-II^+^CD11c^+^ cells in spleens. **g** Tabulated flow cytometric data for CD4^+^Foxp3^+^ expression in the spleen is shown. **h** Flow cytometry results showing the Tem ratios of CD4^+^ T cells or CD8^+^ T cells in spleens. *p < 0.05, **p < 0.01, ***p < 0.001

The spleen, the largest immune organ, plays a critical role in systemic immunity [36–38], and the lymphocytes isolated from it were used here to evaluate the immune response of Nano^PCPT+PIMDQ^. The results demonstrated that Nano^PCPT+PIMDQ^ effectively boosted the beneficial immune function of the spleen. Increased CD8^+^ T and CD4^+^ T cell numbers, along with elevated GrB and IFN-γ respectively expression in CD8^+^ T and CD4^+^ T cells, suggested a cytolytic role (Fig. 7a–e). CD103^+^ DCs are a core subgroup in antigen-presenting cells to promote the production of tumor antigen-specific CD8^+^ T cells, and Fig. 7f showed that administration of Nano^PCPT+PIMDQ^ generated more CD103^+^ DCs than other treatments. The costimulatory molecule CD86 and the antigen-presenting molecules MHC-I and MHC-II on the surface of DCs verified their activation in the spleen. The number of Treg cells (CD4^+^Foxp3^+^ T cells) were also decreased in mice receiving CPT-containing treatment (Fig. 7g). Fortunately, we also observed the formation of effector memory T cells (Tem, CD44^+^CD62L^−^ T cells) gated on CD4^+^ T and CD8^+^ T cells in spleens upon the Nano^PCPT+PIMDQ^ group (Fig. 7h). These results demonstrated that Nano^PCPT+PIMDQ^ effectively primed the immune response after administration and subsequently promoted antitumor effects.

**Fig. 8:**
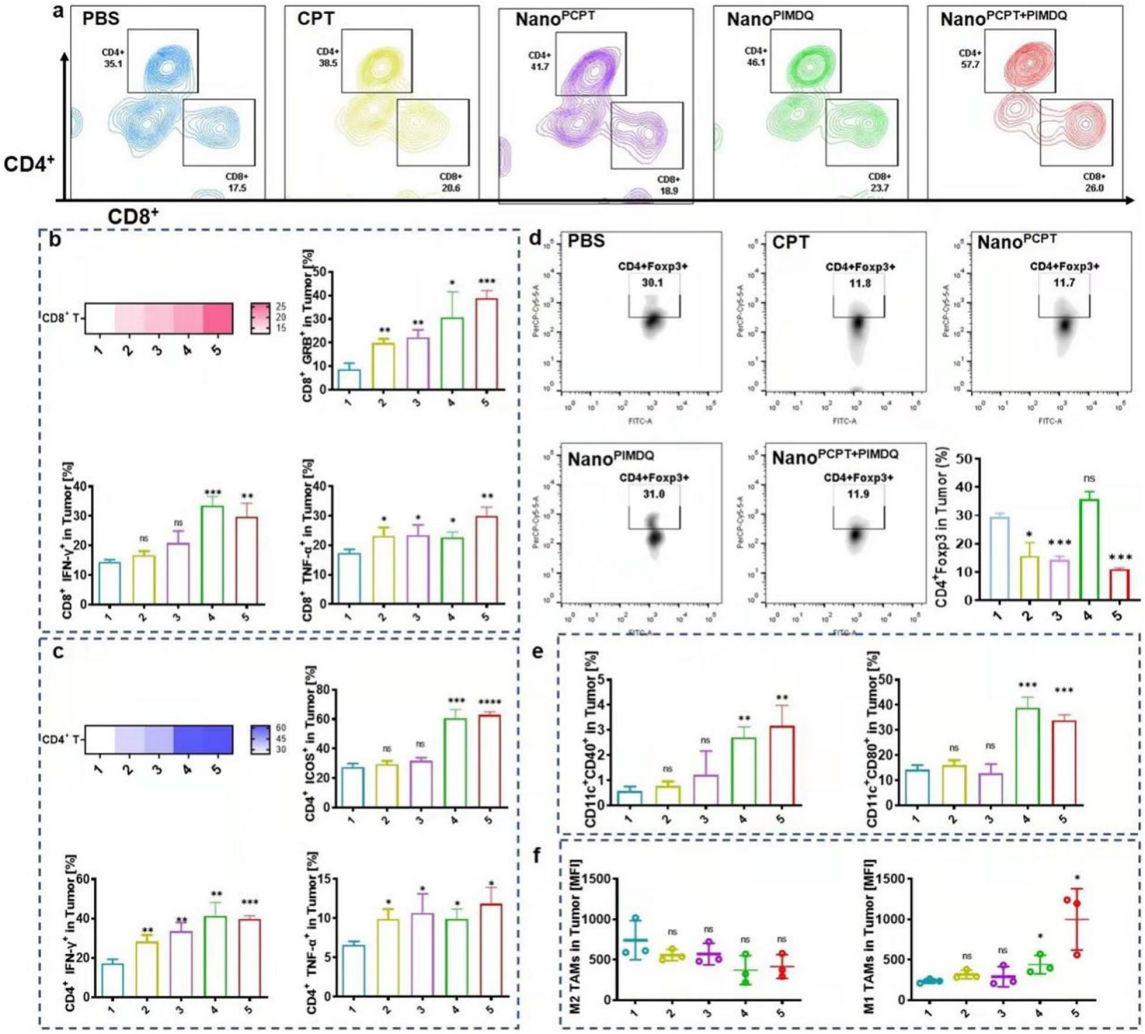
Nano^PCPT+PIMDQ^ enhanced antitumor immunity in Tumors. **a**–**f** Flow cytometry quantification of lymphocytes within tumors from the mice treated with different formulbations. **a** Percentage of CD8^+^ and CD4^+^ T cells gating on CD3^+^ cells harvested in tumors (n = 3). **b** The frequency of CD8^+^ T cells and the expression of GrB, TNF-α, IFN-γ in CD8^+^ T cells (n = 3). **c** The frequency of CD4^+^ T cells and the expression of ICOS, TNF-α, IFN-γ in CD4^+^ T cells (n = 3). **d** Tabulated flow cytometric data for CD4^+^Foxp3^+^ expression in tumor is shown. **e** Flow cytometry analysis of CD11c^+^ CD40^+^, CD11c^+^ CD80^+^ cells in tumors. **f** Flow cytometry analysis of M2 TAMs (CD206), M1 TAMs (iNOS) in tumors. *p < 0.05, **p < 0.01, ***p < 0.001

To investigate the immunocomponent within tumors, we performed a large tumor model (> 200 mm^3^) and pretreated with different formulations via intratumoral injection. Compared with other groups, Nano^PCPT+PIMDQ^ treatment elevated the infiltration of CD8^+^ T cells within tumors. The expression or secretion of GrB, TNF-α, IFN-γ on CD8^+^ T cells were upregulated, demonstrating their activation (Fig. 8a, b). Meanwhile, CD4^+^ T cells proportion were also elevated after treatment with Nano^PCPT+PIMDQ^ and the expression or secretion of ICOS, IFN-γ and TNF-α were upregulated (Fig. 8c). Treg cells are an essential subset of immunosuppressive cells. Foxp3, as the master gene of Treg cells, is required for immunosuppressive function. The accumulation of Foxp3^+^ Treg cells is linked to disease progression and immunosuppression and may alter CD8^+^ T-cell activity [39]. Hence, cancer immunotherapy may be improved by specifically inhibiting Treg cells [40]. As shown in Fig. 8d, the number of Treg cells (CD4^+^Foxp3^+^ T cells) decreased in tumors in mice receiving the CPT-containing treatment, which was in line with earlier research [9]. Subsequently, CD11c antibody was used to label DC to investigate their activation status. Nano^PCPT+PIMDQ^ treatment increased the expression of CD40 and CD80 in comparison with other groups (Fig. 8e). Additionally, Nano^PCPT+PIMDQ^ induced the re-polarization of TAMs based on the downregulation of M2 TAMs and upregulation of M1 TAMs (Fig. 8f). These results indicated that Nano^PCPT+PIMDQ^ hold promise for triggering robust antitumor immune response within tumors.

## Conclusions

In summary, we developed a sequential pH/GSH-responsive triblock polymeric nanomicelle system that codelivers the chemotherapeutic drug CPT and the TLR7/8 agonist IMDQ, inducing amplified chemoimmunotherapy of colorectal cancer. First, Nano^PCPT+PIMDQ^ responding to the tumor acidic microenvironment achieved charge reversal and dispersed into smaller micelles, consequently increasing intracellular uptake and improving the penetration of Nano^PCPT+PIMDQ^ inside tumor tissue. Subsequently, GSH-triggered CPT was released from PCPT in tumor cells. In addition to the chemotherapeutic killing effect, CPT lowered the number of Treg cells (CD4^+^Foxp3^+^) in TDLNs and spleens, allowing the infiltration of cytotoxic T cells into tumors. The exposed PIMDQ remodeled M2 macrophages into M1 macrophages and activated DCs to prime T cells, which further stimulated tumor-specific immune responses. In the CT26 colon tumor model, we showed that Nano^PCPT+PIMDQ^ markedly inhibited the growth of primary and distal tumors. As a consequence, our strategy provides a promising nanoplatform for the codelivery of specific drugs to orchestrate chemoimmunotherapy.

## Materials and methods

### Materials

4-Cyano-4-(dodecylsulfanylthiocarbonyl) sulfanyl pentanoic acid (DCT), N,N′-dicyclohexylcarbodiimide (DCC), acetone oxime, monomethoxy PEG (Mw = 5000 Da), and 2,2′-azobis(2-methylpropionitrile) (AIBN) were purchased from Sigma–Aldrich. 2-Hydroxyethyl disulfide was purchased from Macklin, and 4-(N,N-dimethylamino) pyridine (DMAP) was purchased from Heowns. Triphosgene, methacryl chloride, N-ethylethanolamine, propyl bromide, and camptothecin were obtained from Energy Chemical. 5(6)-TAMRA cadaverine (Rho) was purchased from Haoran Biological Technology Co., Ltd. Cyanine 5.5 amine was purchased from Lumiprobe. 4′,6-Diamidino-2-phenylindole (DAPI) was purchased from Beyotime Biotechnology Co., Ltd. Cell counting kit-8 (CCK-8) was purchased from Beijing Solarbio Technology Co., Ltd. An Annexin V-FITC/PI apoptosis detection kit was purchased from Meilun Biological Technology Co., Ltd. (Dalian, China). 1-(4-(Aminomethyl)benzyl)-2-butyl-1H-imidazo[4,5-c] quinoline-4-amine (IMDQ) was provided by Nanjing Aikon Chemical Ltd. Anti-CD11c, anti-CD86, anti-CD80, anti-CD40, anti-GrB, anti-TNF-α, anti-CD3e, anti-IFN-γ, anti-MHC-I, anti-MHC-II, anti-CD103, anti-F4/80, anti-CD206, anti-iNOS, anti-CD4, anti-CD8, anti-Foxp3, anti-CD44, anti-CD62L were purchased from Biolegeng (American).

### Cell lines

A murine colorectal cell line (CT26) and macrophages were obtained from the School of Pharmacy, Shandong University. CT26 cells were cultured with RPMI-1640 complete growth medium supplemented with 10% fetal bovine serum and 2% penicillin/streptomycin and incubated at 37 °C with 5% CO_2_ saturation. Macrophages were cultured with DMEM complete growth medium supplemented with 10% fetal bovine serum and incubated at 37 °C with 5% CO_2_ saturation. Bone marrow-derived DCs (BMDCs) were obtained from the bone marrow cells of five-week-old male C57BL/6 mice and cultured in 1640 containing recombinant mouse granulocyte–macrophage colony stimulating factor (GM-CSF) and IL-4.

### Animals

Healthy male BALB/c mice (3–5 weeks old) were purchased from Beijing Huafukang Biotechnology Co., Ltd. The Medical Animal Test Center at Shandong University provided male C57BL/6 mice (5–7 weeks old).

### Synthesis of *N*-ethyl-*N*-propylethanolamine

Synthesis of *N*-ethyl-*N*-propylethanolamine: After dissolving N-ethylethanolamine (27.42 g, 0.3 mol), sodium carbonate (47.7 g, 0.45 mol), and propyl bromide (44.28 g, 0.36 mol) in ethanol (100 mL), the mixture was heated to 80 ℃ for 24 h. The mixture was filtered, and the obtained filtrate was concentrated, extracted with DCM, and evaporated under reduced pressure to obtain the product (Additional file 1: Fig. S2). ^1^HNMR (400 MHz, CDCl_3_, δ): 3.53 (t, J = 5.5 Hz, 2H), 2.56 (p, J = 7.1, 6.2 Hz, 4H), 2.42 (dd, J = 8.6, 6.5 Hz, 2H), 1.46 (p, J = 7.4 Hz, 2H), 1.02 (t, J = 7.2 Hz, 3H), 0.89 (t, J = 7.4 Hz, 3H).

### Synthesis of *N*-ethyl-*N*-propyl-aminoethanol methacrylate (EPEMA)

EPEMA was synthesized according to the reported literature with some modifications [41]. After dissolving N-ethyl-*N*-propylethanolamine (3 g, 22.9 mmol) and triethylamine (2.317 g, 22.9 mmol) in acetonitrile (20 mL), the mixture was cooled to 0 ℃. Methacryl chloride (2.394 g, 22.9 mmol) was added dropwise. The mixture was stirred at 0 ℃ for 2 h and at room temperature for another 12 h. The mixture was filtered, and the obtained filtrate was extracted with DCM and evaporated under reduced pressure to give the monomer EPEMA (Additional file 1: Fig. S3). ^1^HNMR (400 MHz, CDCl_3_, δ): 6.09 (t, J = 1.4 Hz, 1H), 5.60–5.51 (m, 1H), 4.20 (t, J = 6.2 Hz, 2H), 2.75 (t, J = 6.3 Hz, 2H), 2.58 (q, J = 7.1 Hz, 2H), 2.53–2.39 (m, 2H), 1.94 (t, J = 1.3 Hz, 3H), 1.51–1.40 (m, 2H), 1.03 (t, J = 7.2 Hz, 3H), 0.87 (t, J = 7.4 Hz, 3H).

### Synthesis of acrylyl acetone oxime (AA)

AA was synthesized according to the reported literature [42]. First, acetone oxime (3.98 g, 54.4 mmol) was dissolved in Milli-Q water (32.5 mL) and cooled to 0 ℃ in an ice bath. Acryloyl chloride (5 g, 55.2 mmol) was added slowly into the mixture at 0 ℃. Then, the reaction mixture was stirred at room temperature for 1 h. Next, the layers were separated, and the aqueous phase was extracted with DCM (3 × 50 mL). After being concentrated under reduced pressure, the organic phase was washed with saturated NaHCO_3_ aqueous solution (3 × 50 mL) and then water (50 mL). The organic phase was dried over Na_2_SO_4_, and the obtained filtrate was evaporated in vacuo to afford AA (Additional file 1: Fig. S4), which was used directly without purification. ^1^HNMR (400 MHz, CDCl_3_, δ): 6.51 (d, J = 17.3 Hz, 1H), 6.20 (dd, J = 17.3, 10.6 Hz, 1H), 5.90 (d, J = 10.6 Hz, 1H), 2.07 (s, 3H), 2.02 (s, 3H).

### Synthesis of OH-2S-CPT

To synthesize OH-2S-CPT, we first synthesized 2-[(2-hydroxyethyl)dithio]ethyl-2-methyl-2-propenoate as reported in the literature [43]. Briefly, 2-hydroxyethyl disulfide (1290 mg, 8.36 mmol) and TEA (845.9 mg, 8.36 mmol) were dissolved in tetrahydrofuran (THF) (20 mL) and cooled to 0 ℃ in an ice bath. Methacryl chloride (873.87 mg, 8.36 mmol) was added slowly to the mixture at 0 ℃. The mixture was stirred at 0 ℃ for 2 h and at room temperature overnight. The mixture was filtered, and the obtained filtrate was extracted with EtOAc and concentrated under reduced pressure to afford a lightly yellow oily liquid. Second, CPT (1000 mg, 2.87 mmol), DMAP (1402.8 mg, 11.48 mmol), and triphosgene (340.74 mg, 1.15 mmol) were dissolved in anhydrous DCM and stirred at room temperature for 0.5 h. 2-[(2-Hydroxyethyl)dithio]ethyl-2-methyl-2-propenoate (701.74 mg, 3.157 mmol) in anhydrous DCM was added slowly to the above reaction solution and then continuously stirred overnight. Subsequently, removal of the solvent under reduced pressure and column chromatography on silica gel yielded the pure product. ^1^HNMR (400 MHz, DMSO-d6, δ): δ 8.69 (s, 1H), 8.14 (t, J = 9.2 Hz, 2H), 7.86 (ddd, J = 8.4, 6.9, 1.4 Hz, 1H), 7.72 (t, J = 7.4 Hz, 1H), 6.01–5.93 (m, 1H), 5.64 (q, J = 1.7 Hz, 1H), 5.52 (d, J = 2.0 Hz, 2H), 5.30 (d, J = 3.2 Hz, 2H), 4.40–4.22 (m, 4H), 3.10–2.90 (m, 4H), 2.17 (dq, J = 10.3, 7.1 Hz, 2H), 1.81 (d, J = 1.3 Hz, 3H), 0.92 (t, J = 7.4 Hz, 3H) (Additional file 1: Fig. S5).

### Synthesis of PEG-DCT

The macro-CTA PEG-DCT was synthesized as previously reported [44]. DCT (162.68 mg, 0.403 mmol), DMAP (49.234 mg, 0.403 mmol), and DCC (83.15 mg, 0.403 mmol) were dissolved in dichloromethane (DCM) (30 mL). PEG (1.55 g, 0.31 mmol) dissolved in DCM (10 mL) was added to the above mixture. The mixture was stirred at room temperature for 36 h. The mixture was filtered, and then, the obtained filtrate was concentrated and purified by triple precipitation in cold ether. The product was then dried under vacuum overnight to obtain a pale yellow powder (Additional file 1: Fig. S6).

### Synthesis of diblock polymer PEG-PEPEMA

PEG-PEPEMA was synthesized by the reversible addition-fragmentation chain transfer (RAFT) polymerization technique. Briefly, PEG-DCT (270.24 mg, 0.050 mmol), EPEMA (500 mg, 2.51 mmol), and AIBN (2.47 mg, 0.015 mmol) were dissolved in dioxane and placed into Schlenk tubes. After four freeze–pump–thaw cycles, the mixture was heated to 70 °C to initiate polymerization and stirred for 48 h. The solution was cooled and purified by dialysis against water. PEG-PEPEMA was obtained by lyophilization.^1^HNMR spectroscopy was used to determine the monomer conversion (Additional file 1: Fig. S7).

### Synthesis of the triblock polymer prodrug PEG-PEPEMA-PCPT (PCPT)

The polymerization of PEG-PEPEMA-PCPT also used RAFT polymerization. PEG-PEPEMA (356 mg, 0.024 mmol), OH-2S-CPT (432.8 mg, 0.725 mmol), and AIBN (1.2 mg, 0.007 mmol) were dissolved in dioxane and dimethyl sulfoxide (DMSO) (volume ratio = 1:1) and placed into Schlenk tubes. After four freeze–pump–thaw cycles, the mixture was heated to 80 °C to initiate polymerization and stirred for 48 h. The solution was cooled, and the polymer prodrug was purified by triple precipitation in cold ether to afford the polymer prodrug (Additional file 1: Fig. S8). The drug grafting rate was determined by UV–vis analysis (Additional file 1: Fig. S11).

### Synthesis of triblock polymer PEG-PEPEMA-PAA

The polymerization of PEG-PEPEMA-PAA also used RAFT polymerization. PEG-PEPEMA (642 mg, 0.044 mmol), AA (166.4 mg, 1.309 mmol), and AIBN (2.15 mg, 0.013 mmol) were dissolved in dioxane and placed into a Schlenk tube. After four freeze–pump–thaw cycles, the mixture was heated to 75 °C to initiate polymerization and stirred for 48 h. The solution was cooled and purified by dialysis against water. PEG-PEPEMA-PAA was obtained by lyophilization (Additional file 1: Fig. S9).

### Synthesis of PEG-PEPEMA-PAA-PIMDQ (PIMDQ)

To connect IMDQ, we employed the amino group on IMDQ and the PAA on the polymer to conduct a substitution process. PEG-PEPEMA-PAA (20 mg, 0.00115 mmol), IMDQ (5.4 mg, 0.0125 mmol), and TEA (3.79 mg, 0.0374 mmol) were dissolved in dioxane and stirred at 50 ℃ for 48 h under N_2_ protection. The solution was cooled and dialyzed against water overnight. A white powder was obtained by lyophilization. The IMDQ grafting rate was determined by UV–vis analysis (Additional file 1: Fig. S12).

### Synthesis of PEG-PEPEMA-PAA^Rho^

To fluorescently label the polymer, we employed the amino group on Rho and the PAA on the polymer to conduct a substitution process. In short, PEG-PEPEMA-PAA (0.0034 mmol), Rho (0.67 μmol), and TEA (0.036 mmol) were dissolved in dioxane and stirred at 50 ℃ for 48 h under N_2_ protection. The solution was cooled and dialyzed against water for 24 h to remove free Rho. A pink power was obtained by lyophilization.

### Synthesis of PEG-PEPEMA-PAA^cy5.5^

To fluorescently label the polymer, we employed the amino group on Cy 5.5 and the PAA on the polymer to conduct a substitution process. In short, PEG-PEPEMA-PAA (0.003 mmol), NH2-Cy5.5 (0.7 μmol), and TEA (0.038 mmol) were dissolved in dioxane and stirred at 50 ℃ for 48 h under N_2_ protection. The solution was cooled and dialyzed against water for 24 h to remove free Cy5.5. A green powder was obtained by lyophilization.

### Self-assembly of PEG-PEPEMA-PCPT and PEG-PEPEMA-PIMDQ triblock polymer mixture nanomicelles (Nano.^PCPT+PIMDQ^)

After dissolving PEG-PEPEMA-PCPT (11.25 mg) and PEG-PEPEMA-PIMDQ (1.7 mg) in dimethyl sulfoxide (0.3 mL), the above solution was dropped into Milli-Q water (3 mL) under ultrasound. The mixture was sonicated for 30 min to create nanomicelles. To remove DMSO, the solution was dialyzed against deionized water overnight. Finally, the stock solution was diluted to 3.2 mg·mL^−1^ and stored in the dark at 4 °C for further experiments.

### Characterization of Nano^PCPT+PIMDQ^

We diluted the micelle stock solution with various pH buffers to investigate the pH effect on micelles. After overnight incubation at 37 °C, dynamic light scattering (DLS) was used to determine the count rate and zeta potential, and TEM was used to examine the micelle morphology.

### Self-assembly of PEG-PEPEMA-PCPT nanomicelles (Nano.^PCPT^)

Sonication was also used to prepare Nano^PCPT^. After dissolving PEG-PEPEMA-PCPT (60 mg) in DMSO (0.5 mL), the above solution was dropped into Milli-Q water (18 mL) under sonication for 30 min. To remove DMSO, the solution was dialyzed against deionized water overnight. Finally, the stock solution was diluted to 3 mg·mL^−1^ and stored in the dark at 4 °C for further testing.

### Self-assembly of PEG-PEPEMA-PIMDQ nanomicelles (Nano.^PIMDQ^)

After dissolving PEG-PEPEMA-PIMDQ (5 mg) in DMSO (0.1 mL), the above solution was dropped into Milli-Q water (3 mL) under sonication for 30 min. To remove DMSO, the solution was dialyzed against deionized water overnight. Finally, the stock solution was diluted to 1 mg·mL^−1^ and stored in the dark at 4 °C for further testing.

### Preparation of TAMRA-labeled nanomicelles (Nano.^Rho^)

Similarly, PEG-PEPEMA-PTAMRA was dissolved in DMSO and then added dropwise into Milli-Q water under continuous sonication. After sonication for 30 min, the solution was dialyzed against deionized water for 24 h to remove DMSO. Finally, the stock solution (10 mg·mL^−1^) was stored in the dark at 4 °C for further experiments.

### Preparation of Cy5.5-labeled nanomicelles (Nano.^cy5.5^)

The Cy5.5-labeled polymer was dissolved in DMSO and then added dropwise into Milli-Q water under constant sonication. After 30 min, the solution was dialyzed against deionized water for 24 h using a dialysis bag (3500 Da MWCO) to remove DMSO and obtain Nano^cy5.5^. All the above experiments were carried out under dark conditions.

### Drug grafting rate

According to Lambert–Beer law, under the same experimental conditions, the absorbance (A) is proportional to the concentration (C). The certain concentrations of CPT and IMDQ were used as the controls (C_control_) and absorbance were determined by UV–visible (UV–vis) spectrophotometry at 365 nm and 326 nm, respectively (A_sample_). The drug-grafting content (DGC) were calculated as follows formula:

$$\begin{aligned} {\text{W}}_{{{\text{CPT}}({\text{IMDQ}})}} & = {\text{ V }} \times {\text{ C}}_{{{\text{sample}}}} = {\text{ V }} \times \, \left( {{\text{C}}_{{{\text{control}}}} \times {\text{ A}}_{{{\text{sample}}}} } \right) \, /{\text{A}}_{{{\text{control}}}} ; \\ {\text{DGC }}\left( \% \right) \, & = \, \left( {{\text{W}}_{{{\text{CPT}}({\text{IMDQ}})}} /{\text{ W}}_{{{\text{PEG}} - {\text{PEPEMA}} - {\text{PCPT}}({\text{PEG}} - {\text{PEPEMA}} - {\text{PIMDQ}}}} } \right) \, \times {1}00\% . \\ \end{aligned}$$

The DGC results of CPT and IMDQ were 34.09 ± 0.46%, 13.62 ± 0.19% respectively (Additional file 1: Figs. S11-S12).

### In vitro drug release of CPT triggered by the reducing agent dithiothreitol (DTT)

In a shaking incubator at 37 °C, a Nano^PCPT^ dispersion in pH 7.4 phosphate-buffered saline (PBS) was placed into a dialysis bag (3500 Da MWCO), which was then dialyzed against the buffered medium in the absence or presence of DTT (10 mM). An aliquot of the external medium was withdrawn and replaced with an equal volume of new buffer solution at various periods. HPLC was used to measure CPT concentrations in the media at different time intervals using a 365 nm absorption wavelength.

### Cultivation of tumor spheroids

As reported in the literature, the hanging drop technique was used to prepare tumor spheroids [45]. CT26 cells were resuspended at 400,000·mL^−1^ in 0.25% methylcellulose, and 20 μL of suspension was transferred dropwise to the lid of the 48-well plate, with PBS (200 μL·well^−1^) supplied to each well to maintain the humidity of the hanging drop. After incubating at 37 °C with 5% CO_2_ for 4 days, the tumor spheroids were transferred to a 48-well plate containing fresh culture medium for further study.

### In vitro evaluation of tumor penetration of nanomicelles

The obtained tumor spheroids were transferred to 48-well plates with fresh media (pH 7.4 or 6.5) containing Nano^cy5.5^ (Cy5.5 = 2 μg·mL^−1^). After incubation for 6 h, the tumor spheroids were washed twice with ice-cold PBS and fixed with 1% paraformaldehyde. Subsequently, z-stack images were obtained with a confocal laser scanning microscope.

### In vitro effect of pH on intracellular trafficking of nanomicelles

CT26 cells were plated in a 48-well plate with 100,000 cells per well and incubated for 12 h at 37 °C with 5% CO_2_. The culture media were removed, and the cells were treated with fresh media (pH 7.4 or 6.5) containing Nano^cy5.5^. After 12 h, the supernatant was removed, the cells were separated, washed with cold PBS, and resuspended in PBS, and the sample was measured using flow cytometry.

## In vitro Cytotoxicity Assay by CCK-8

CT26 cells were plated at 5000 cells per well in a 96-well plate and incubated for 12 h. Afterward, the supernatant was removed by pipette aspiration and replaced with a series of media containing free CPT, Nano^PCPT^, and Nano^blank polymer^ at a series of concentrations, followed by incubation for another 24 h. Subsequently, the drug-containing medium was removed by pipette aspiration, replaced with fresh medium, and then incubated for 48 h. Next, CCK-8 (10 μL) was added to each well and incubated for 40 min, and the absorption was measured at 465 nm. The other operations were the same as those for CT26 cells, with the exception of incubating macrophages with 3 μM CPT and Nano^PCPT^ containing the same quantity of CPT.

## In vitro BMDC uptake

Murine BMDCs were obtained and cultured from C57BL/6 mice. BMDCs were cultured with medium containing Nano^Rho^ for 2, 6, 12, 24 h. Afterward, the cells were collected, washed with cold PBS twice, and resuspended in PBS, and the sample was measured using flow cytometry.

## In vitro BMDC activation

To explore the effect of polymer-modified IMDQ on the activation of BMDCs, we incubated BMDCs with different concentrations of free IMDQ and Nano^PIMDQ^ in a 48-well plate for 48 h. Cells were then collected and stained with anti-CD11c, anti-CD86, anti-CD80, anti-MHC-II-BV786, and anti-CD40 to determine the specific DC maturation marker expression by flow cytometry (FACS Celesta) and analyzed using FlowJo software.

## In vitro macrophage repolarization

RAW264.7 macrophage cells were seeded in a 12-well plate at a density of 500,000 cells/well overnight. After adding IL-4 for 24 h to simulate polarization into M2 macrophages, various concentrations of IMDQ and Nano^PIMDQ^ were added for incubation for another 24 h. Cells were then collected and stained with anti-F4/80, anti-CD200R, and anti-iNOS to determine the expression of specific macrophage repolarization markers by flow cytometry (FACS Celesta) and analyzed using FlowJo software.

## IVIS imaging and biodistribution in vivo

CT26-bearing mice were obtained by inoculating 800,000 CT26 cells on the backs of male BALB/c mice. When tumor sizes grew to 100–200 mm^3^, mice were used for biodistribution studies. After Nano^cy5.5^ intratumoral injection, real-time fluorescence images of the anesthetized mice were captured by an in vivo imaging system at different time points (1, 24, 48, 96, and 120 h) to investigate the time-dependent retention of Nano^cy5.5^ in tumors. The mice were sacrificed, and their major organs were excised for in vitro imaging. The excised livers and tumors were used to extract liver and tumor cells for flow cytometry quantification, respectively.

## Subcutaneous Tumor Model Evaluation

CT26 cells (750,000) in PBS were injected subcutaneously into the right back of BALB/c mice (5–7 weeks old), followed three days later by subcutaneous injection of the same density of CT26 cells in the left back. When the proximal tumors grew to 50–60 mm^3^, the mice bearing CT26 tumors were intratumorally injected with five different groups of medicine: 1. PBS; 2. CPT; 3. Nano^PCPT^; 4. Nano^PIMDQ^; and 5. Nano^PCPT+PIMDQ^. CPT was administered at a dose of 10 mg·kg^−1^, while IMDQ was given at a dose of 0.5 mg·kg^−1^ body weight. The mice received a total of four treatments, and they were given drugs every three days. The body weight and tumor volumes were recorded regularly, and the tumor volumes (V) were calculated from the following equation: V = LW^2^/2 (L = length of tumor longest axis; W = length of the axis perpendicular to the longest axis of the tumor). The survival of the mice was closely monitored throughout the experiment.

## In vivo antitumor immune response post-administration

To assess whether our mixed nanomicelles could elicit an improved immune response, mice from each group were dissected after four intratumoral injections. Subsequently, tumor-draining lymph nodes and spleen were removed from the mice to prepare a single immune cell suspension. Anti-CD3, anti-CD4, anti-CD8, anti-GrB, anti-TNF-α, and anti-IFN-γ were labeled to measure cytotoxic T-cell activation in TDLNs, and anti-CD4, anti-CD8, anti-CD3, and anti-GrB, anti-IFN-γ were labeled to measure cytotoxic T-cell activation in the spleen. Anti-CD11c, anti-MHC-I, and anti-MHC-II were labeled to assess DC cell maturation in TDLNs, and anti-CD86, anti-CD103, anti-MHC-I, and anti-MHC-II were labeled to assess DC cell maturation in spleens. Anti-F4/80, anti-CD206, and anti-iNOS were labeled to evaluate macrophage repolarization from M2 to M1 in TDLNs.

Finally, the cells were analyzed by flow cytometry (FACS Celesta) and FlowJo software.

## Antitumor immune response in vivo at the end of treatment

We prepared single-cell suspensions from the tumors, tumor-draining lymph nodes and spleen for immunological analysis and evaluation. Single-cell suspensions of tumors, lymph nodes and spleens were stained with anti-CD4, anti-Foxp3 for the analysis of Treg cells. A single-cell suspension of spleens was stained with anti-CD44 and anti-CD62L to analyze immune memory and the effector memory T cells were based on the CD44^+^ CD62L^−^ phenotype.

## Anti-tumor immune response within tumors

To investigate the immune response within tumors, we performed a large tumor model (> 200 mm^3^) and pretreated with different formulations via intratumoral injection. After treatment, single-cell suspensions collected from tumors were labeled with anti-CD3e, anti-CD4, anti-CD8 to evaluate T cell infiltration; anti-GrB, anti-TNF-α, anti-IFN-γ, and anti-ICOS to evaluate the activation of T cells; anti-CD4 and anti-Foxp3 to evaluate Treg cells; anti-CD11c, anti-CD40, and anti-CD80 to evaluate DCs maturation; anti-F4/80, anti-CD206, and anti-iNOS to evaluate macrophage repolarization. These cells were performed via flow cytometry (FACS Celesta) and analyzed via FlowJo software.

## Immunohistochemical analysis

Main organs and tumors were taken and fixed with 4% formaldehyde before being stained with hematoxylin and eosin (H&E) for an in vivo safety investigation, and tumors were further stained with Ki67 and TUNEL for further research.

## Statistical analysis

Student’s *t test* was used for statistical analysis, and the results are expressed as the means ± SD. Statistical significance: *p < 0.05, **p < 0.01, ***p < 0.001. (GraphPad Software, CA, USA).

## Supplementary Information

**Additional file 1.** Sequential acid/reduction response of triblock copolymeric nanomicelles to release camptothecin and toll-like receptor 7/8 agonist for orchestrated chemoimmunotherapy. **Figure S1.** The synthetic route of EPEMA, AA, OH-2S-CPT. **Figure S2.** 1H-NMR spectrum of 2-(N-Ethyl-N-propyl) ethanol amine. **Figure S3.** 1H-NMR spectrum of EPEMA. **Figure S4.** 1H-NMR spectrum of AA. **Figure S5.** 1H-NMR spectrum of OH-2S-CPT. **Figure S6.** 1H-NMR spectrum of PEG-DCT. **Figure S7.** 1H-NMR spectrum of PEG-PEPEMA. **Figure S8.** 1H-NMR spectrum of PEG-PEPEMA-PCPT. **Figure S9.** 1H-NMR spectrum of PEG-PEPEMA-PAA. **Figure S10.** 1H-NMR spectrum of PEG-PEPEMA-PIMDQ. **Figure S11.** UV-vis spectrum of PEG-PEPEMA-PCPT. **Figure S12.** UV-vis spectrum of PEG-PEPEMA-PIMDQ. **Figure S13.** DTT-triggered CPT release from Nano PCPT in vitro, at the release medium with different pH values. **Figure S14.** CT26 cytotoxicity of PEG-PEPEMA-PAA polymer detected by CCK-8. **Figure S15.** In vitro uptake of nanomicelles by BMDCs at different times (n=3). **Figure S16.** The flow cytometric images of in vitro BMDCs maturation. **Figure S17.** H&E (200×, bar 100 μm) staining of major organs slides after the final treatment in different preparation groups. **Figure S18.** Representative flow cytometric analysis images of CD11c+ MHC-II+ in spleens.

## Data Availability

All data generated or analyzed during this study are included in this published article.

## Abbreviations

RAFT: Reversible Addition-Fragmentation Chain Transfer
AA: Acryloyl acetone oxime
EPEMA: 2- (N-ethyl-N-propyl amino) ethyl methacrylate
IMDQ: 1-(4- (Aminomethyl) benzyl)-2-butyl-1H-imidazo[4,5-c] quinoline-4-amine
CPT: Camptothecin
PCPT: PEG-PEPEMA-PCPT
PIMDQ: PEG-PEPEMA-PIMDQ
AA: Acryloyl acetone oxime
EPEMA: 2-(*N*-Ethyl-*N*-propyl amino) ethyl methacrylate
OH-2S-CPT: Reduction-responsive CPT monomer
PEG: Monomethoxy poly (ethylene glycol)
DCT: 4-Cyano-4-(dodecylsulfanylthiocarbonyl) sulfanyl pentanoic acid
AIBN: 2,2′-Azobis(2-methylpropionitrile)
DCC: N,N′-Dicyclohexylcarbodiimide
DMAP: 4-(N,N-dimethylamino) pyridine
IMQ: Imiquimod
TEA: Triethylamine
Rho: 5(6)-TAMRA cadaverine
cy5.5: Cyanine 5.5 amine
DCM: Dichloromethane
DMSO: Dimethyl sulfoxide
DMSO-*d6*: Dimethyl sulfoxide-d6
CDCl_3_: Trichloromethane-d
EtOAc: Ethyl acetate
DP: Degree of polymerization
GSH: Glutathione
DTT: Dithiothreitol
DLS: Dynamic light scattering
UV–vis: Ultraviolet and visible spectrophotometry
CLSM: Confocal laser scanning microscopy
CCK-8: Cell counting KIT-8
MCSs: 3D multicellular spheroids
TAMs: Tumor-associated macrophages
BMDCs: Bone marrow-derived dendritic cells
TDLNs: Tumor draining lymph nodes
TIME: Tumor immunosuppressive microenvironment
Foxp3: Forkhead box protein 3
TLR: Toll-like receptors
CTLs: Cytotoxic T lymphocytes
Treg: Regulatory T cells
TUNEL: Terminal deoxynucleotidyl transferase-mediated dUTP-biotin nick end labeling
PDI: Polydispersity index
TEM: Transmission electron microscope
DCs: Dendritic cells
DMEM: Dulbecco’s modified eagle medium
DMSO: Dimethyl sulfoxide
H&E: Hematoxylin and eosin
DAPI: 4′,6-Diamidino-2-phenylindole
