# Supplementary material for: Sequential acid/reduction response of triblock copolymeric nanomicelles to release camptothecin and toll-like receptor 7/8 agonist for orchestrated chemoimmunotherapy
Source: J Nanobiotechnology. 2022 Aug 11;20:369. doi: 10.1186/s12951-022-01577-5 (PMC9367092; doi:10.1186/s12951-022-01577-5)
Supplement: Supplementary file 1 — Additional file 1. Sequential acid/reduction response of triblock copolymeric nanomicelles to release camptothecin and toll-like receptor 7/8 agonist for orchestrated chemoimmunotherapy. Figure S1. The synthetic route of EPEMA, AA, OH-2S-CPT. Figure S2. 1H-NMR spectrum of 2-(N-Ethyl-N-propyl) ethanol amine. Figure S3. 1H-NMR spectrum of EPEMA. Figure S4. 1H-NMR spectrum of AA. Figure S5. 1H-NMR spectrum of OH-2S-CPT. Figure S6. 1H-NMR spectrum of PEG-DCT. Figure S7. 1H-NMR spectrum of PEG-PEPEMA. Figure S8. 1H-NMR spectrum of PEG-PEPEMA-PCPT. Figure S9. 1H-NMR spectrum of PEG-PEPEMA-PAA. Figure S10. 1H-NMR spectrum of PEG-PEPEMA-PIMDQ. Figure S11. UV-vis spectrum of PEG-PEPEMA-PCPT. Figure S12. UV-vis spectrum of PEG-PEPEMA-PIMDQ. Figure S13. DTT-triggered CPT release from Nano PCPT in vitro, at the release medium with different pH values. Figure S14. CT26 cytotoxicity of PEG-PEPEMA-PAA polymer detected by CCK-8. Figure S15. In vitro uptake of nanomicelles by BMDCs at different times (n=3). Figure S16. The flow cytometric images of in vitro BMDCs maturation. Figure S17. H&E (200×, bar 100 μm) staining of major organs slides after the final treatment in different preparation groups. Figure S18. Representative flow cytometric analysis images of CD11c+ MHC-II+ in spleens. [file 12951_2022_1577_MOESM1_ESM.docx]

***Additional file***

**Title**

**Sequential Acid/Reduction Response of Triblock Copolymeric Nanomicelles to Release Camptothecin and Toll-Like Receptor 7/8 Agonist for Orchestrated Chemoimmunotherapy**

**Authors**:

Xiaoyan Ge 1#, Yanyun Hao 1#, Hui Li 1, Huajun Zhao 2, Yang Liu 1, Yutong Liu 1, Xia Li 1, Hongfei Chen 1, Jing Zou 1, Shiying Zhang 1, Lingling Huang 1, Gang Shan 3, Zhiyue Zhang 1*

**Affiliations:**

*1*Department of Pharmaceutics, Key Laboratory of Chemical Biology (Ministry of Education), School of Pharmaceutical Sciences, Cheeloo College of Medicine, Shandong University, 44 Wenhuaxi Road, Jinan, Shandong Province 250012, P. R. China.

*2*Institute of Immunopharmaceutical Sciences, School of Pharmaceutical Sciences, Cheeloo College of Medicine, Shandong University, Jinan, Shandong Province 250012, P. R. China.

*3*Department of Medicinal Chemistry, School of Pharmaceutical Sciences, Cheeloo College of Medicine, Shandong University, Jinan, Shandong 250012, P. R. China.

**#**These authors contributed equally to this work.

***Corresponding authors:**Prof. Dr. Zhiyue Zhang, E-mail: zhiyue.zhang@sdu.edu.cn


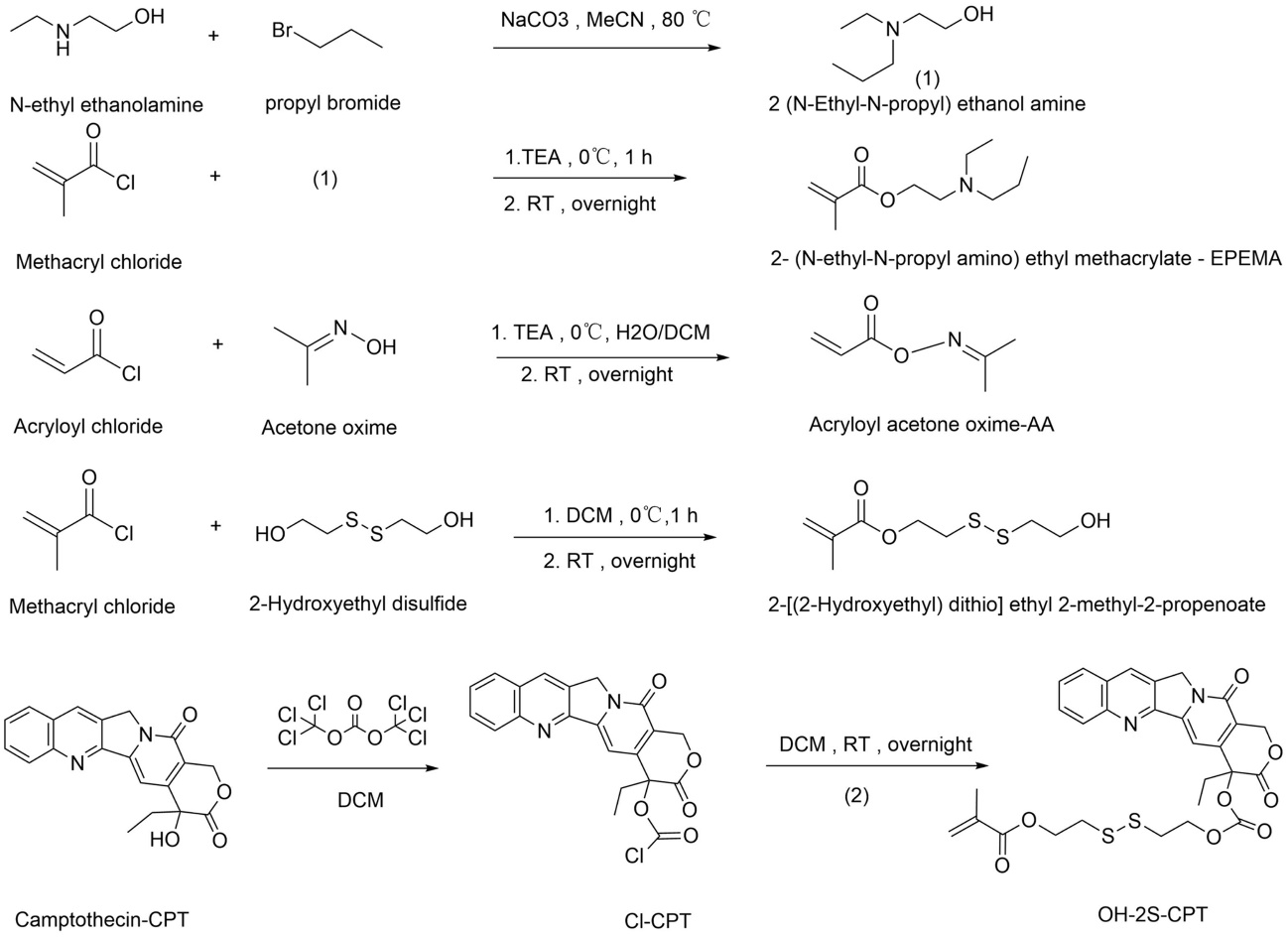


**Fig S1.** The synthetic route of EPEMA, AA, OH-2S-CPT.


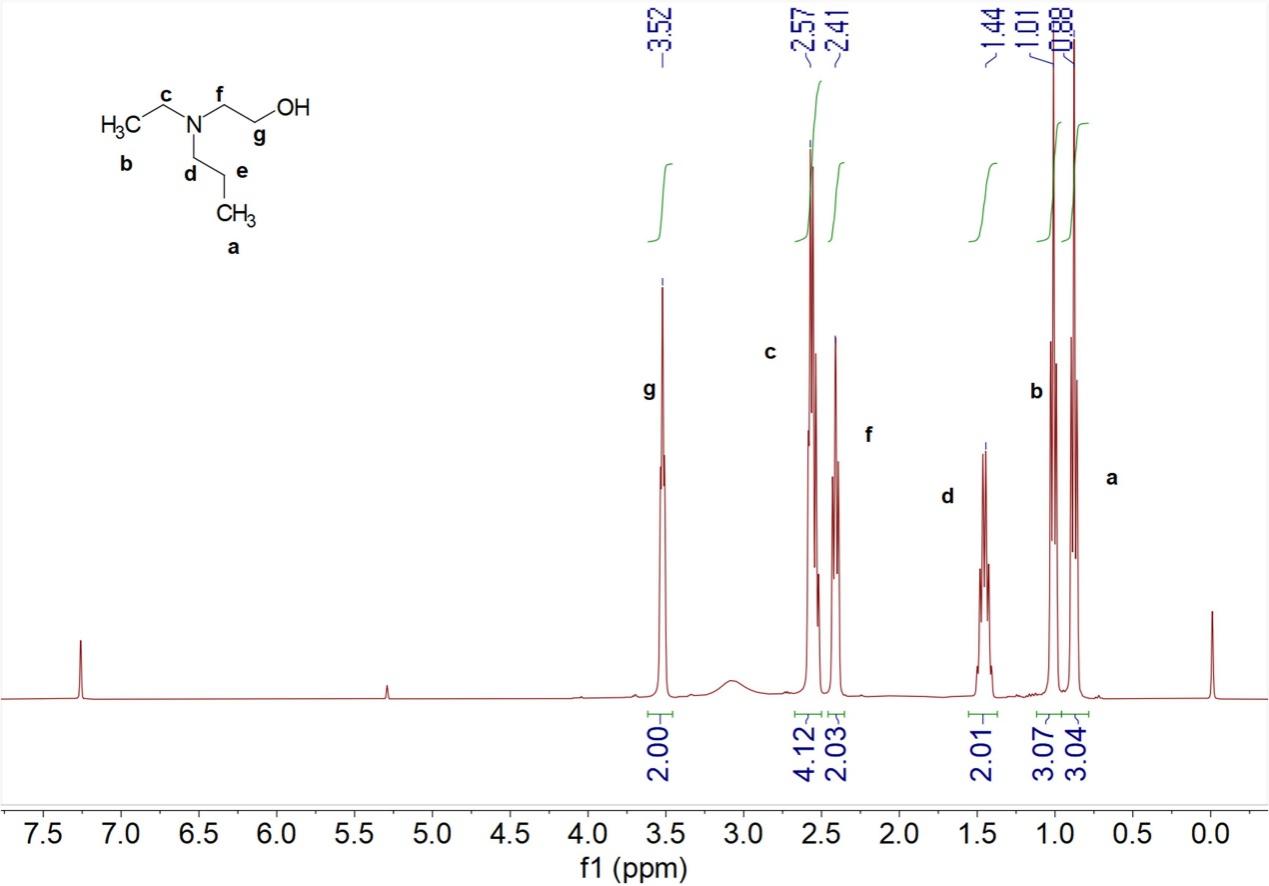


**Fig S2.** ^1^H-NMR spectrum of 2-(N-Ethyl-N-propyl) ethanol amine.


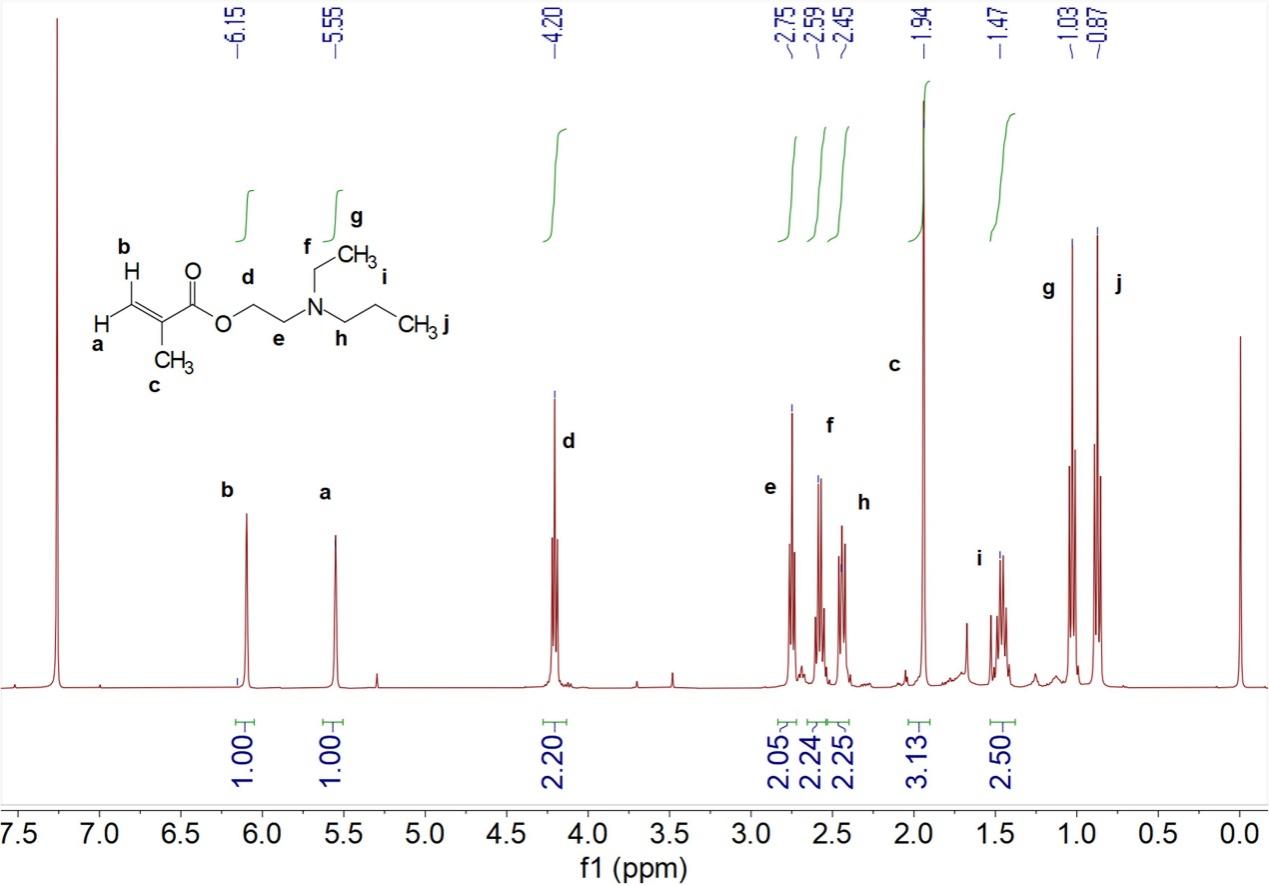


**Fig S3.** ^1^H-NMR spectrum of EPEMA.


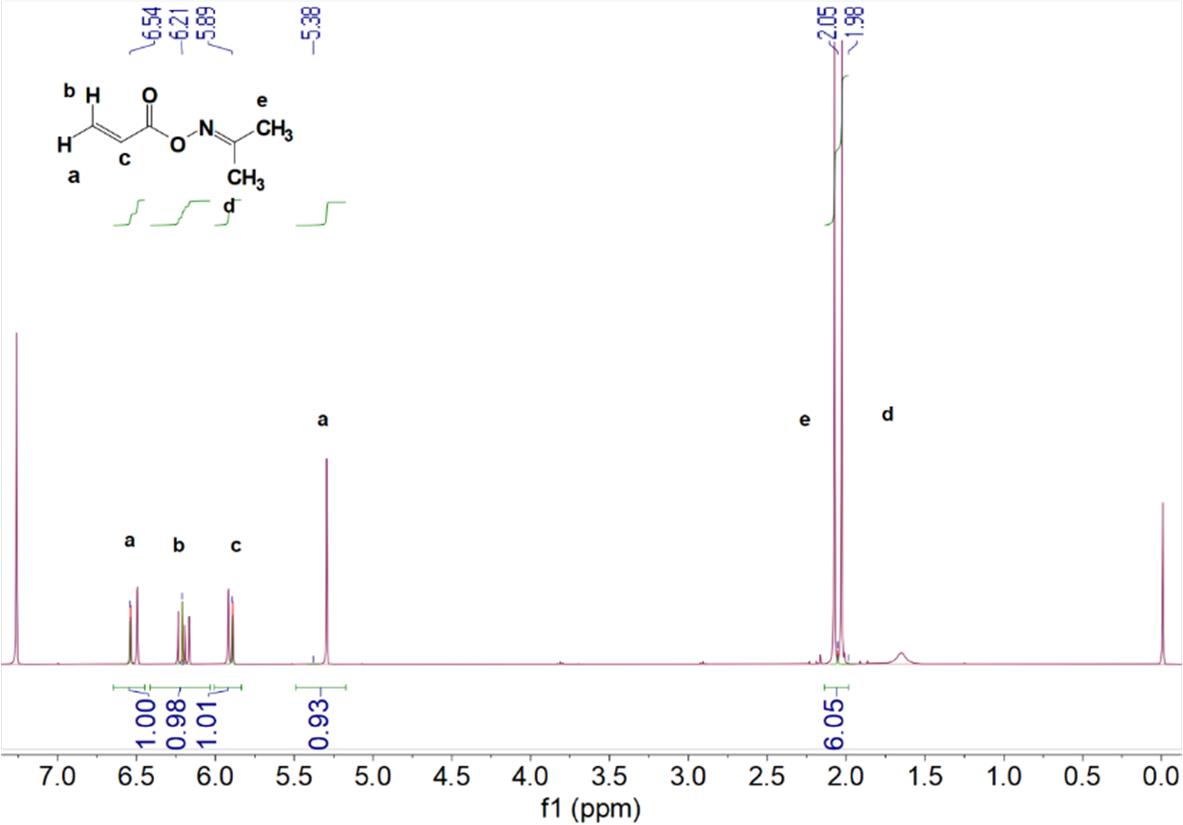


**Fig S4.** ^1^H-NMR spectrum of AA.

**
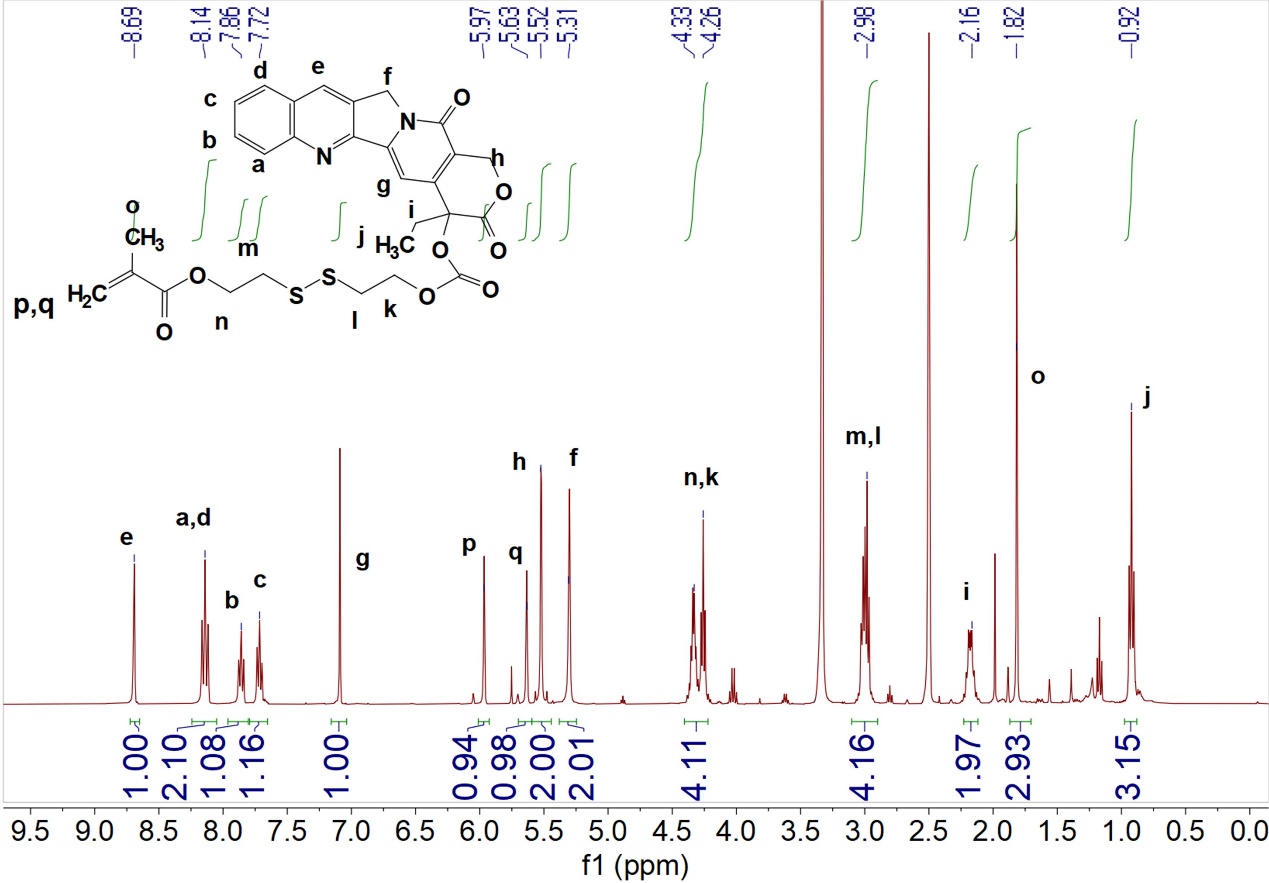
**

**Fig S5.** ^1^H-NMR spectrum of OH-2S-CPT.


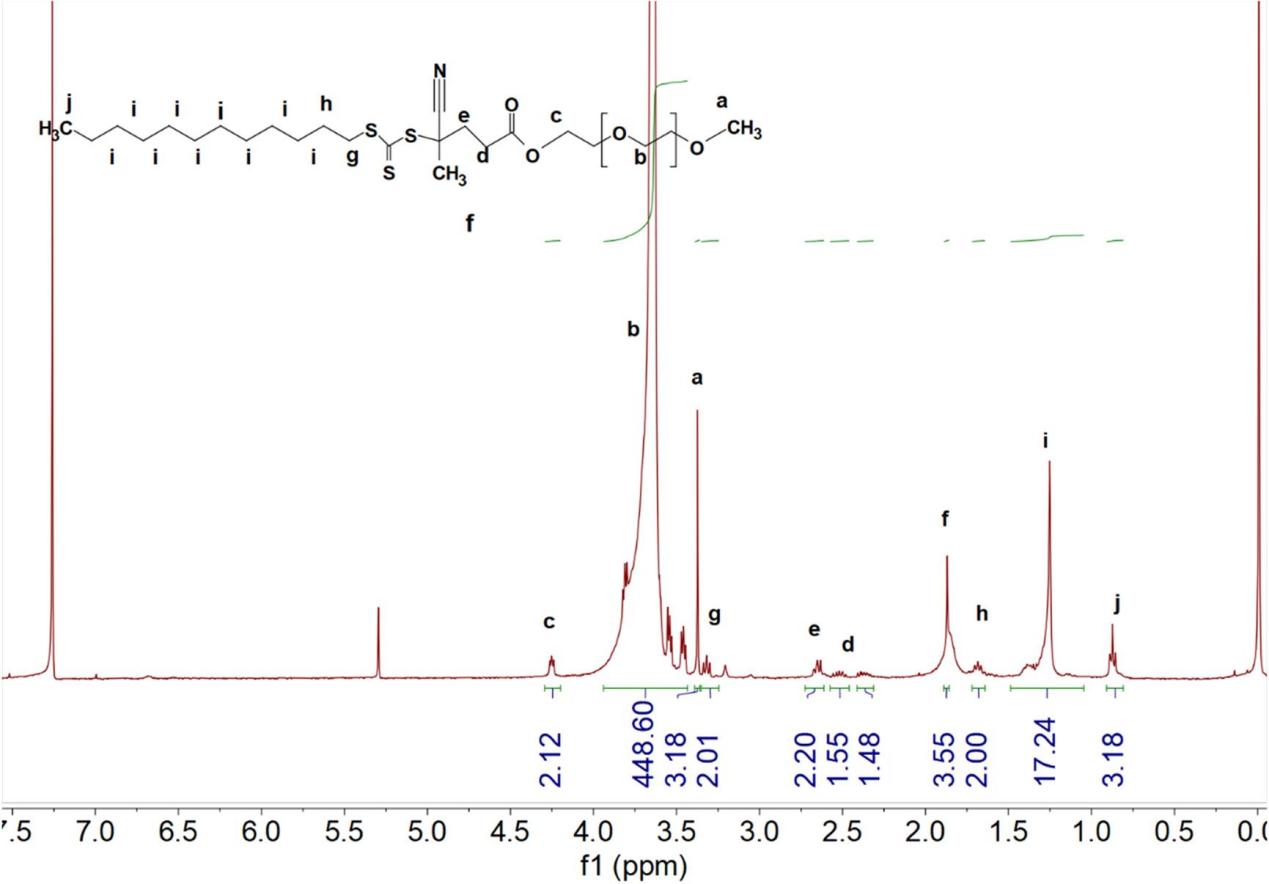


**Fig S6.** ^1^H-NMR spectrum of PEG-DCT.


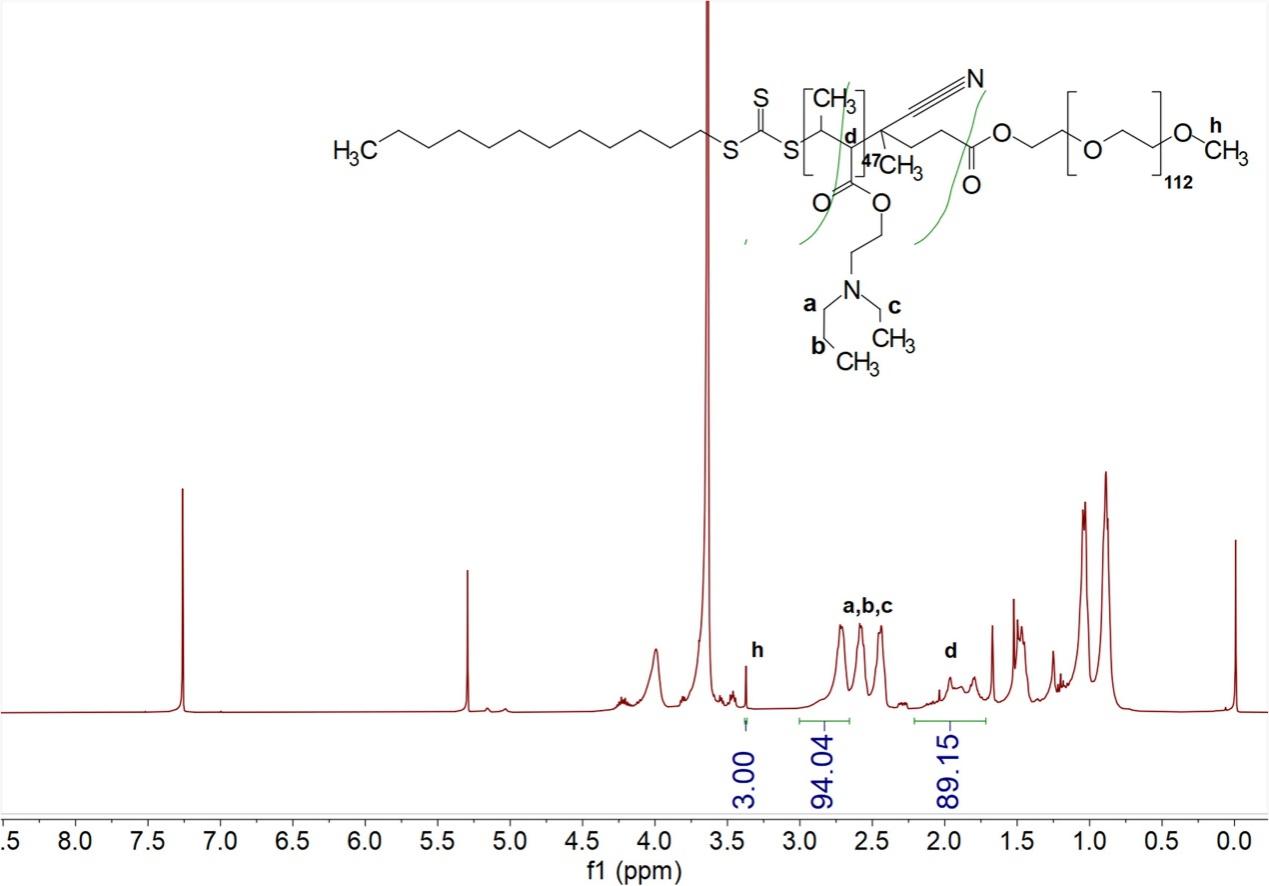


**Fig S7.** ^1^H-NMR spectrum of PEG-PEPEMA.


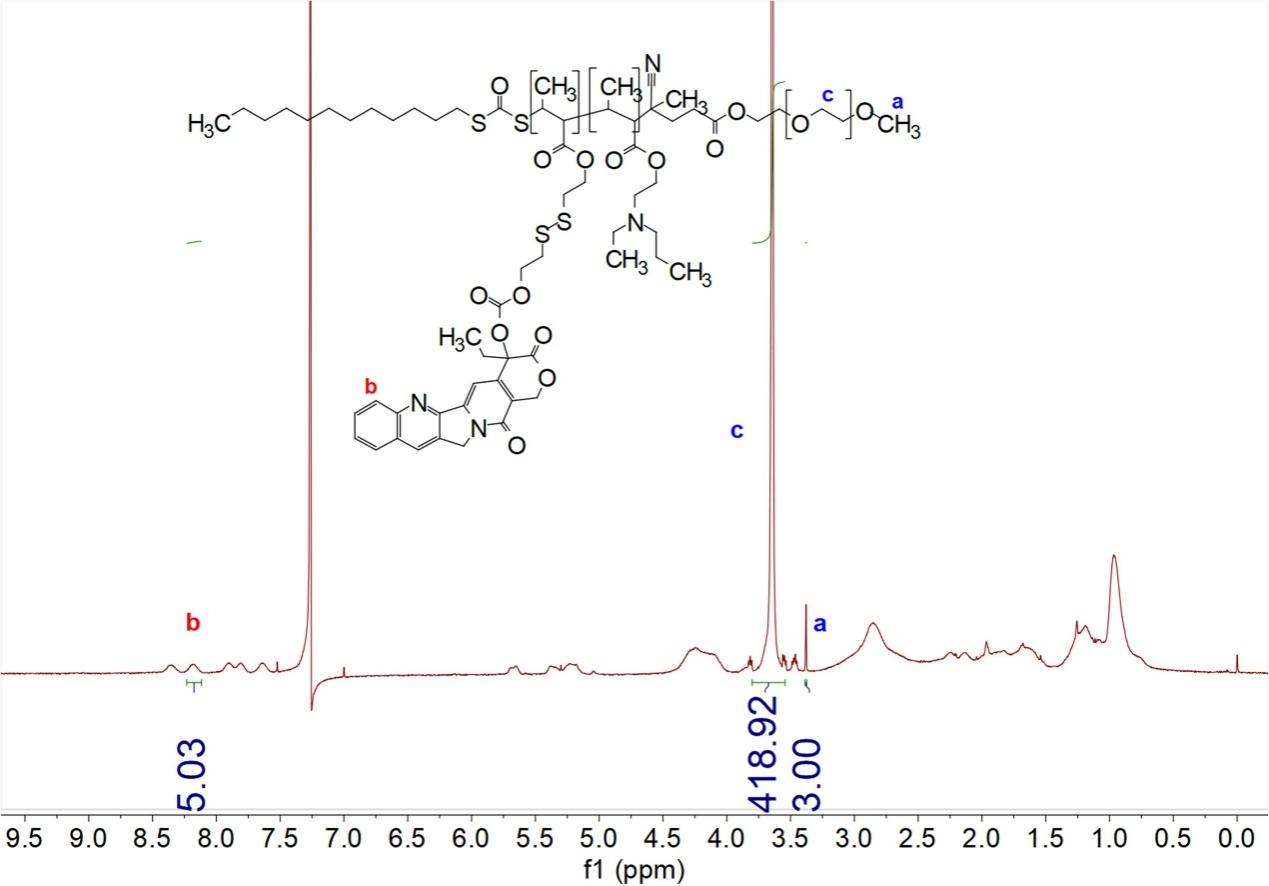


**Fig S8.** ^1^H-NMR spectrum of PEG-PEPEMA-PCPT.


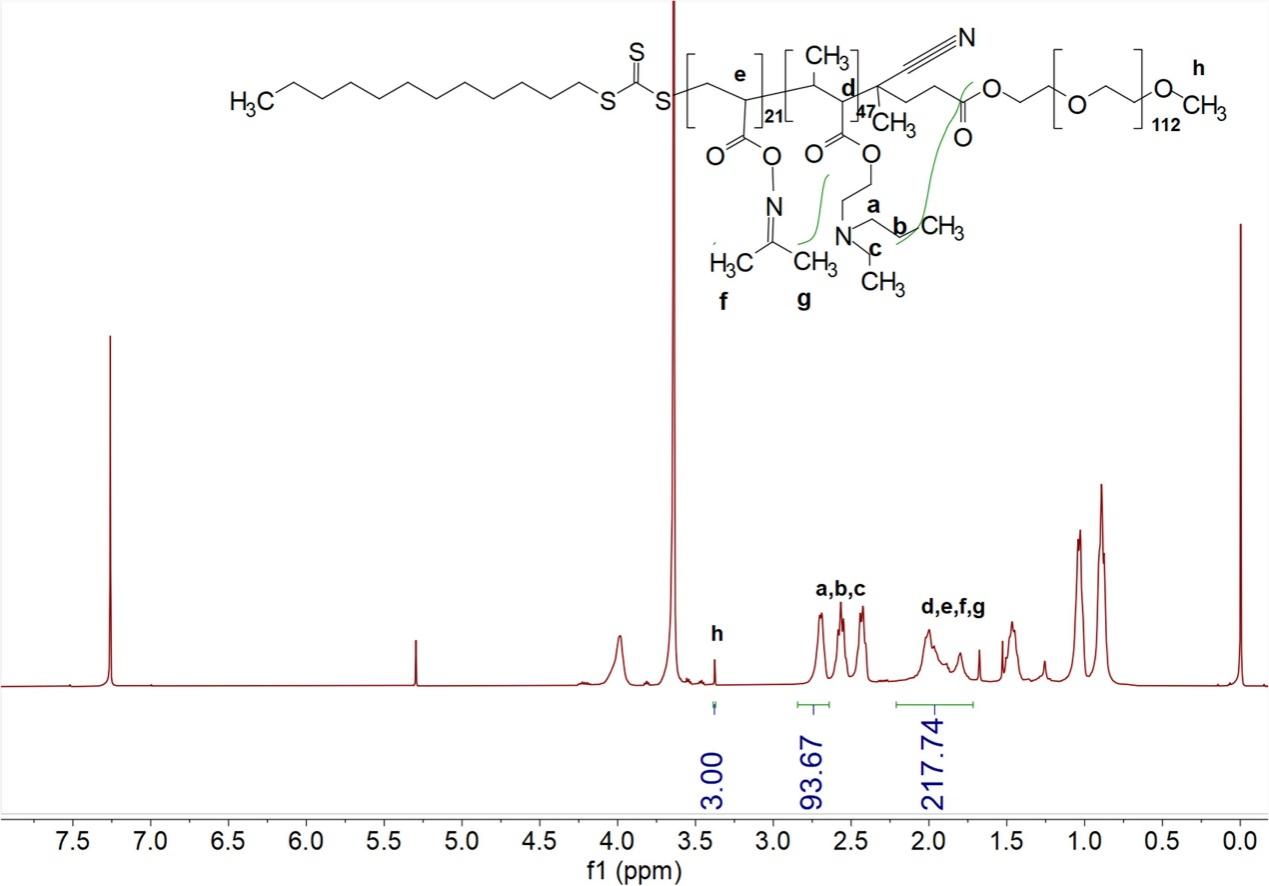


**Fig S9.** ^1^H-NMR spectrum of PEG-PEPEMA-PAA.


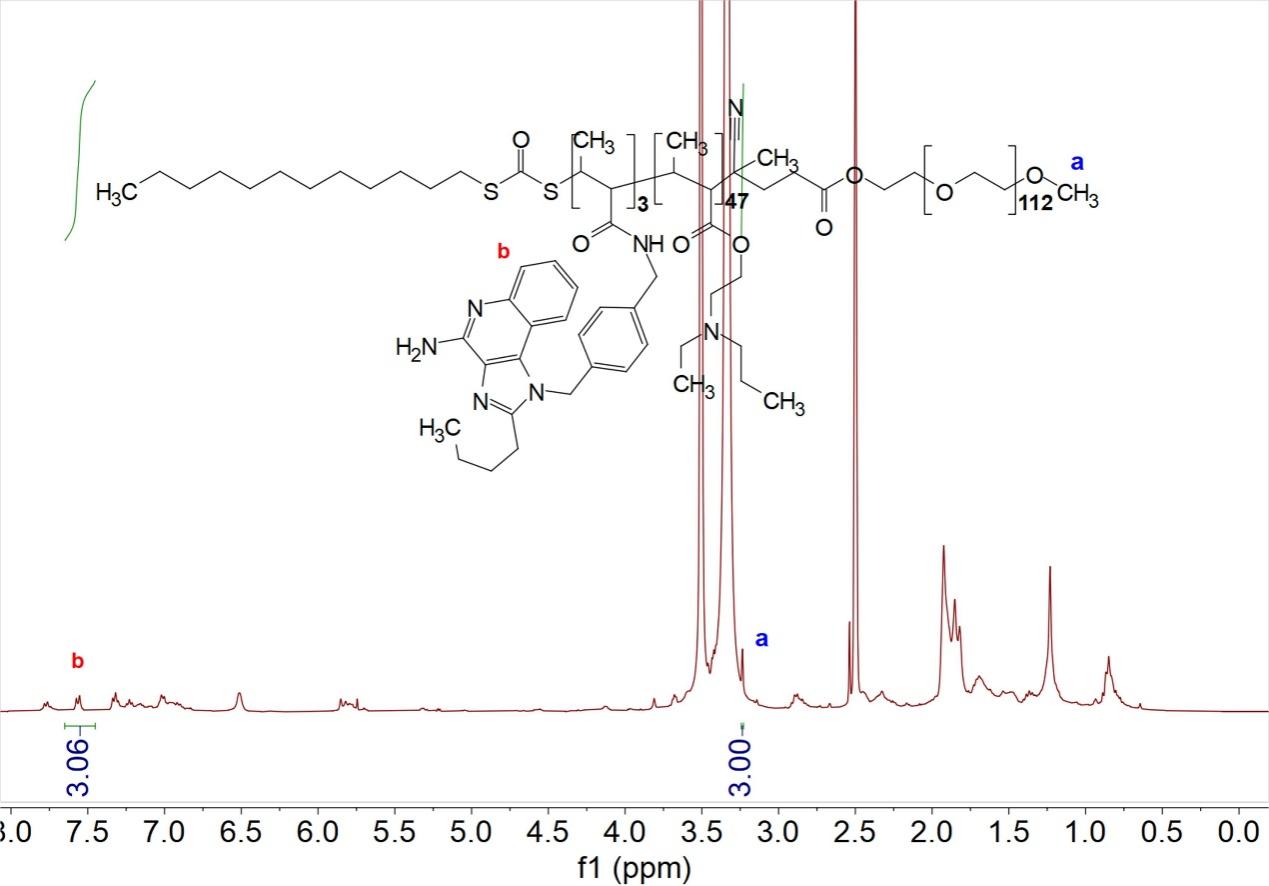


**Fig S10.** ^1^H-NMR spectrum of PEG-PEPEMA-PIMDQ.


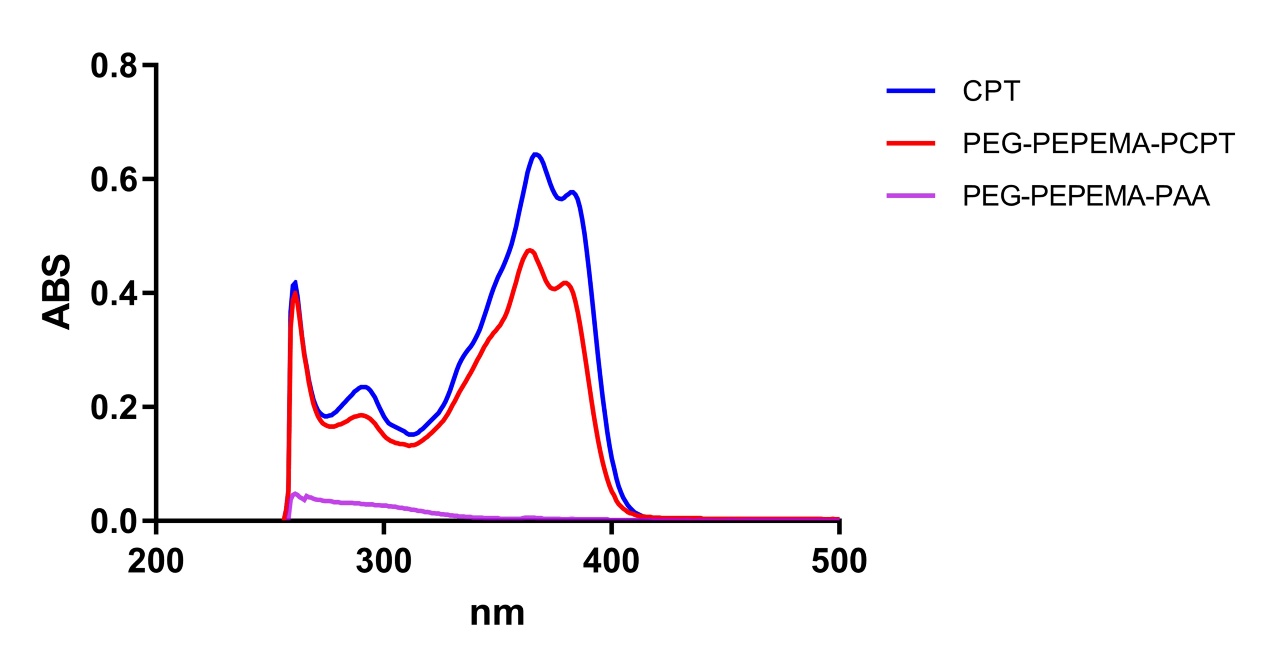


**Fig S11**. UV-vis spectrum of PEG-PEPEMA-PCPT.


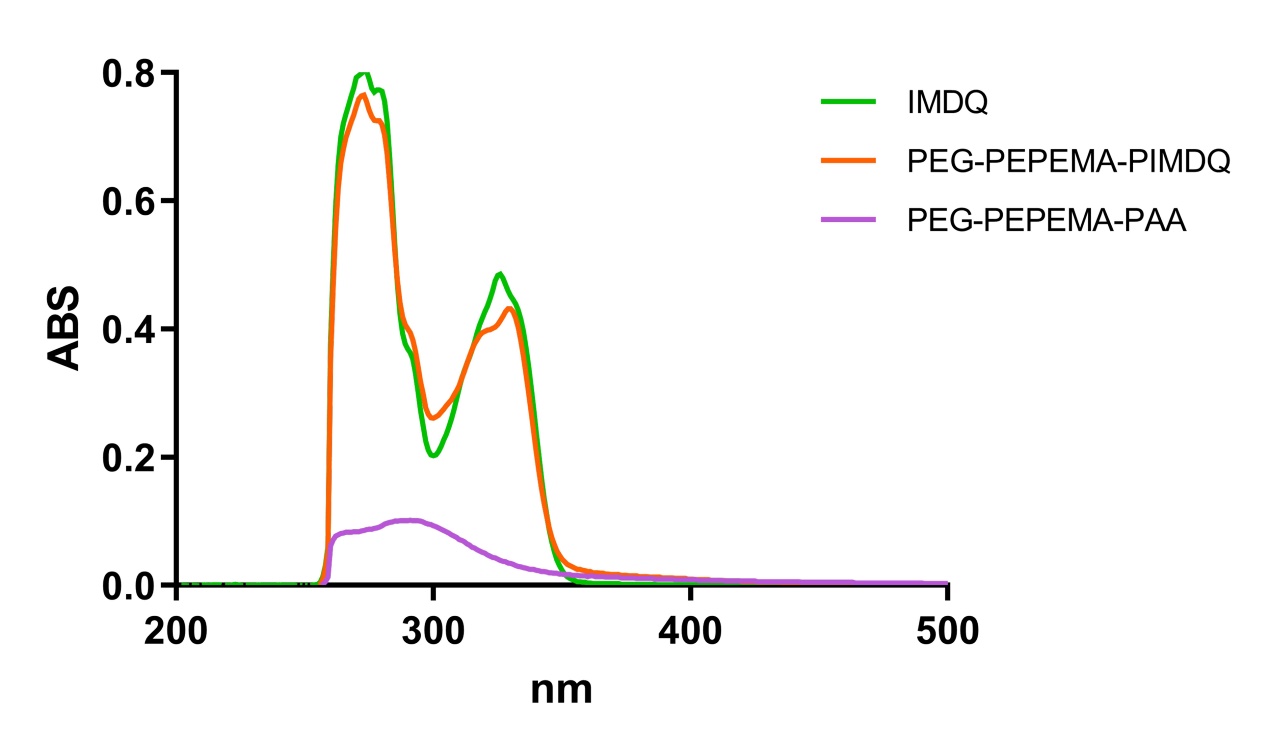


**Fig S12.** UV-vis spectrum of PEG-PEPEMA-PIMDQ.


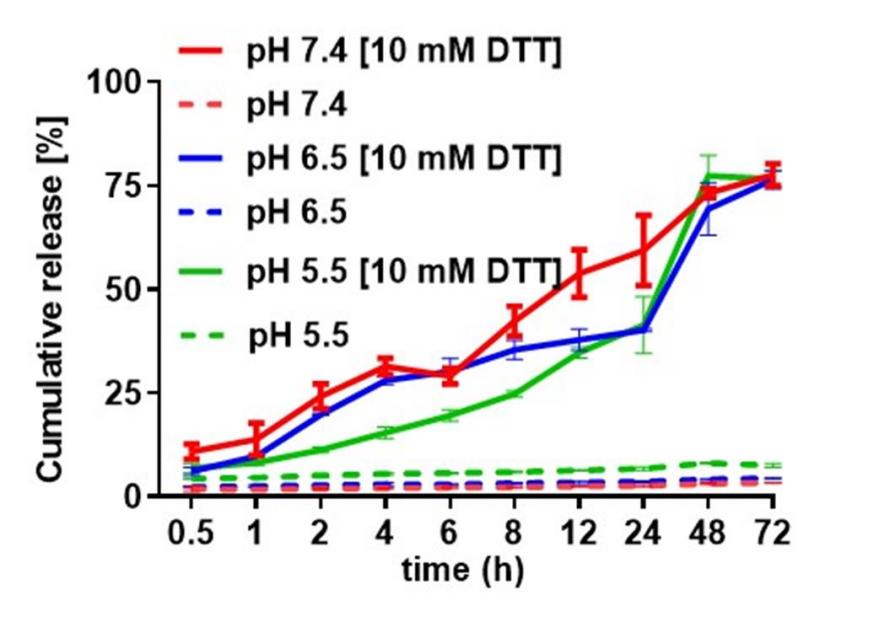


**Fig S13.** DTT-triggered CPT release from Nano^PCPT^ *in vitro*, at the release medium with different pH values.


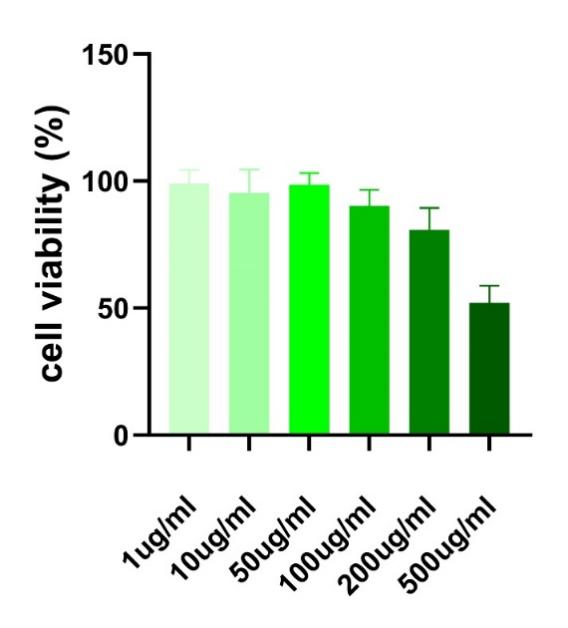


**Fig S14**. CT26 cytotoxicity of PEG-PEPEMA-PAA polymer detected by CCK-8.


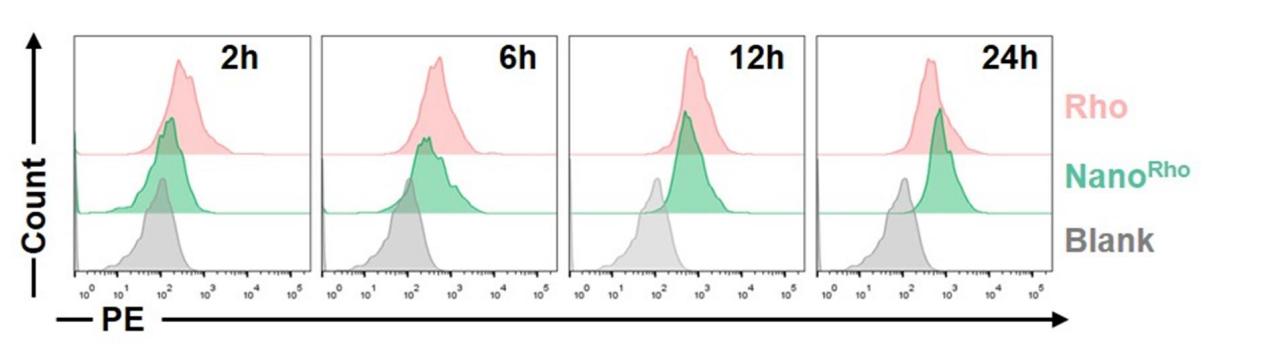


**Fig S15**. *In vitro* uptake of nanomicelles by BMDCs at different times (n=3).


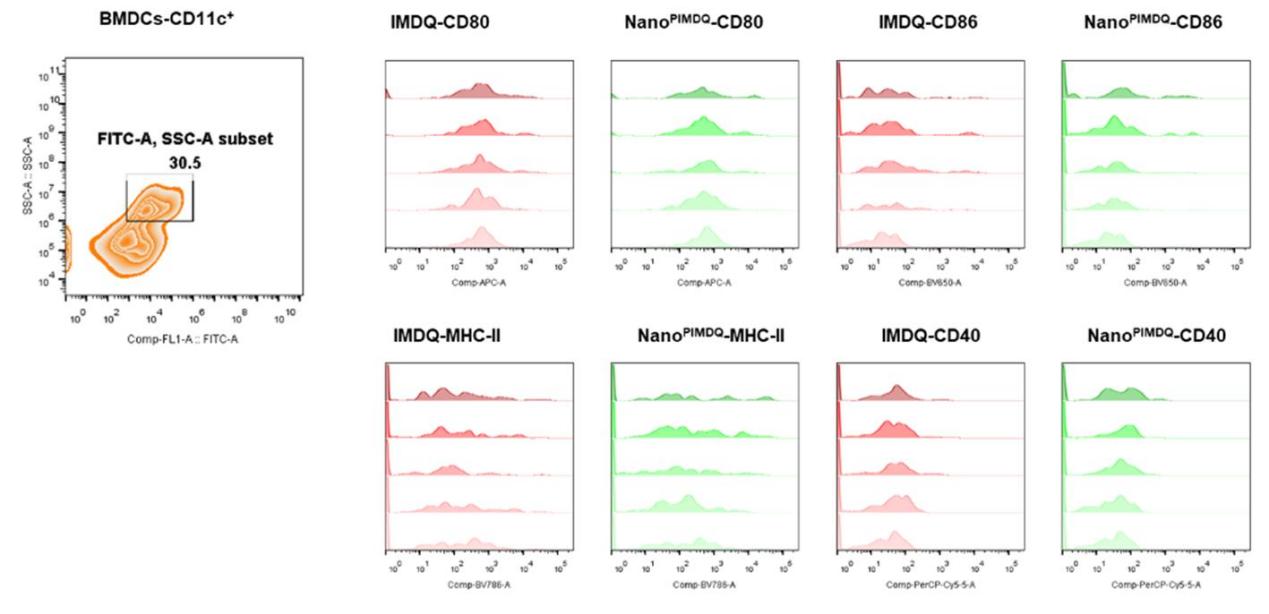


**Fig S16**. The flow cytometric images of *in vitro* BMDCs maturation.


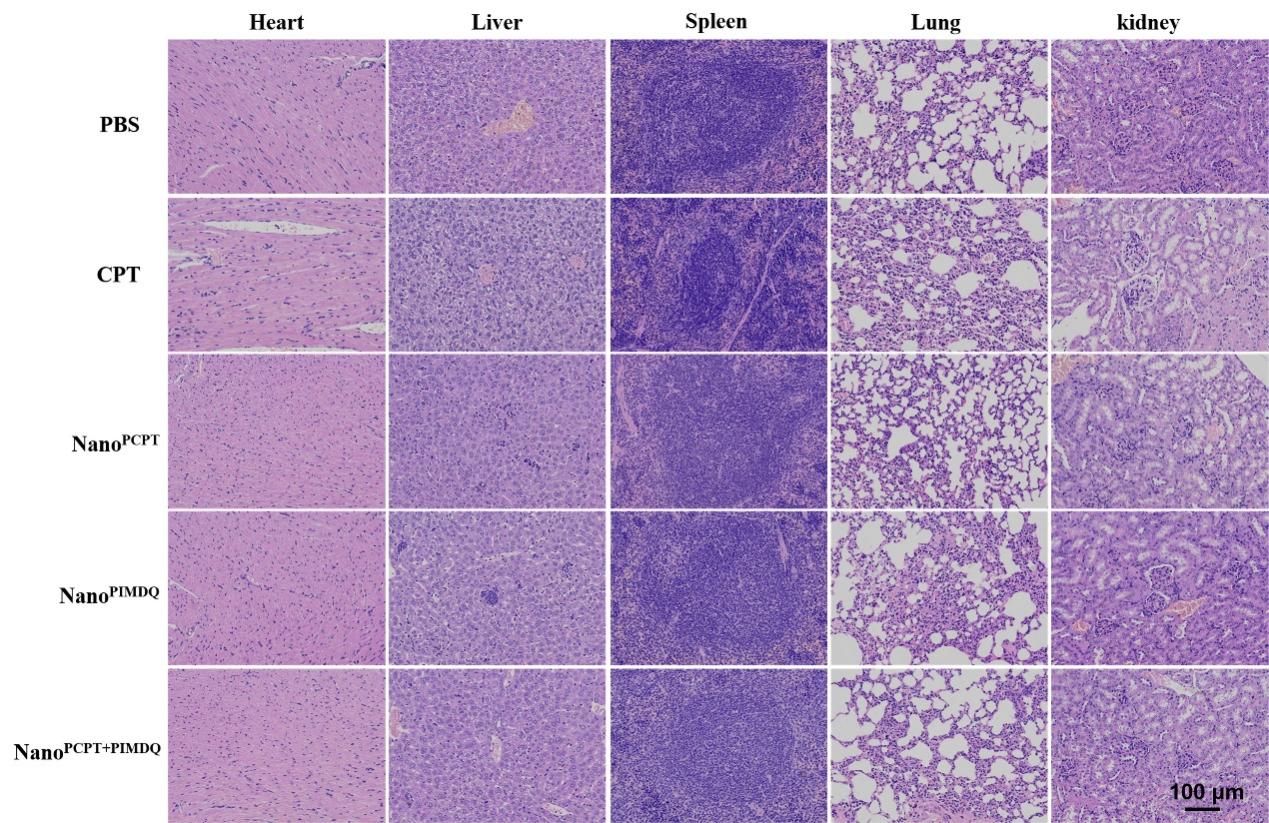


**Fig S17**. H&E (200×, bar 100μm) staining of major organs slides after the final treatment in different preparation groups.


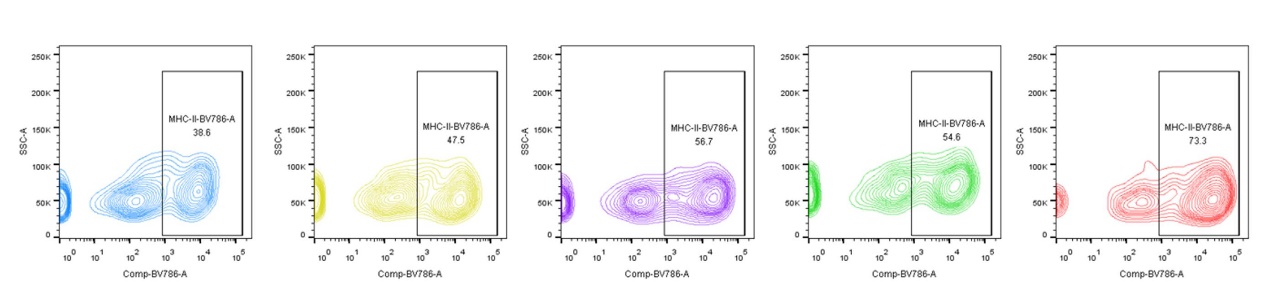


**Fig S18**. Representative flow cytometric analysis images of CD11c^+^ MHC-II^+^ in spleens.
